# Dynophore-Based Approach in Virtual Screening: A Case of Human DNA Topoisomerase IIα

**DOI:** 10.3390/ijms222413474

**Published:** 2021-12-15

**Authors:** Matej Janežič, Katja Valjavec, Kaja Bergant Loboda, Barbara Herlah, Iza Ogris, Mirijam Kozorog, Marjetka Podobnik, Simona Golič Grdadolnik, Gerhard Wolber, Andrej Perdih

**Affiliations:** 1 National Institute of Chemistry, Hajdrihova 19, SI-1000 Ljubljana, Slovenia; matej.janezic@riken.jp (M.J.); katja.valjavec@ki.si (K.V.); kaja.bergantloboda@ki.si (K.B.L.); barbara.herlah@ki.si (B.H.); iza.ogris@ki.si (I.O.); mirijam.kozorog@ki.si (M.K.); marjetka.podobnik@ki.si (M.P.); simona.grdadolnik@ki.si (S.G.G.); 2 Laboratory for Structural Bioinformatics, RIKEN Center for Biosystems Dynamics Research, 1-7-22 Suehiro-cho, Tsurumi-ku, Yokohama 230-0045, Japan; 3 Faculty of Pharmacy, University of Ljubljana, Aškerčeva 7, SI-1000 Ljubljana, Slovenia; 4 Faculty of Medicine, University of Ljubljana, Vrazov trg 2, SI-1000 Ljubljana, Slovenia; 5 Institute of Pharmacy, Freie Universität Berlin, Königin-Luise-Straße 2-4, 14195 Berlin, Germany; gerhard.wolber@fu-berlin.de

**Keywords:** human DNA topoisomerase IIα, catalytic inhibitors, dynophore models, drug design, molecular simulations, cancer research

## Abstract

In this study, we utilized human DNA topoisomerase IIα as a model target to outline a dynophore-based approach to catalytic inhibitor design. Based on MD simulations of a known catalytic inhibitor and the native ATP ligand analog, AMP-PNP, we derived a joint dynophore model that supplements the static structure-based-pharmacophore information with a dynamic component. Subsequently, derived pharmacophore models were employed in a virtual screening campaign of a library of natural compounds. Experimental evaluation identified flavonoid compounds with promising topoisomerase IIα catalytic inhibition and binding studies confirmed interaction with the ATPase domain. We constructed a binding model through docking and extensively investigated it with molecular dynamics MD simulations, essential dynamics, and MM-GBSA free energy calculations, thus reconnecting the new results to the initial dynophore-based screening model. We not only demonstrate a new design strategy that incorporates a dynamic component of molecular recognition, but also highlight new derivates in the established flavonoid class of topoisomerase II inhibitors.

## 1. Introduction

Virtual screening of molecular libraries is now a routine process for which an increasing number of tools are available [1]. Due to the high computational and time costs, the flexibility of proteins is often neglected, even though pioneering reports on their importance were published decades ago [2,3,4]. However, with the increasing affordability of computing power and the development of efficient algorithms, dynamic molecular design methods are becoming more common [5,6]. We have explored the dynamic nature of proteins in the past, from the rudimentary use of multiple structures [7], to analyzing binding pockets and studying the role of water molecules via simulations [8,9].

Broadly, in silico methods of molecular design can be divided into ligand-based and structure-based methods, the latter requiring the structural data of the target molecule. Crystallography, NMR spectroscopy, and electron microscopy have made great strides [10] and the number of structures deposited in the Protein Data Bank has recently surpassed 180.000 entries [11]. Despite great achievements, there are still plenty of therapeutic targets where experimental data are incomplete or absent. In these situations, methods such as homology modeling [12] that can predict 3D protein structures can be exploited [13].

The most omnipresent computational structure-based design method is molecular docking, the beginnings of which can be traced back to the 1970s [14]. Since then, a variety of protocols and scoring functions have been developed, and their complexity and resource requirements vary considerably; it can take from a few seconds to hours to determine the best scoring position of a ligand. Docking results are highly dependent on the initial definition of the active site—from the choice of coordinates to the definition of properties. The definition of binding pockets and the extraction of descriptors for suitable binding partners can also be achieved, for example, by analyzing the thermodynamic properties of water molecules in proteins [9]. As we advance our knowledge in allostery, it is clear that we must look not only to the prominent active sites but at proteins as a whole for possible small molecule interaction hotspots. Hence the emergence of computational methods for mapping allostery [15,16]. 

Ligand-based design methods are based on the knowledge of at least one (potential) binding partner. The binders’ properties are then calculated and serve as a search template to find new binding partners by applying the paradigm “similar molecules should bind in a similar way.” Methods can range from simple substructure searches to similarity searches that incorporate shape and/or electrostatic data, and pharmacophore models or filters from various 2D parameters such as Tanimoto index, logP, etc. [17,18]. Finally, there are also hybrid methods such as structure-based pharmacophores that use both structural and ligand information [18,19]. 

Unfortunately, in virtual screening and molecular design, there is no one-size-fits-all ideal approach due to the diversity of targets and their unique properties. The design of screening protocols is often influenced by familiarity with certain methods or a desire to use the latest method appearing in the literature. When deciding which technique or combination of techniques to use, it is useful to consider all available experimental and computational knowledge of the target and related biological systems as a starting point [20]. From there, it is up to the computational chemist to develop an appropriate protocol. However, when sufficient data are available, one can adjust and fine-tune the screening process [21] using benchmarks such as ROC curves, enrichment factors, and feedback from the performed experiments [22].

Cancer cells exhibit a highly deregulated cell cycle, making DNA topoisomerases, which regulate DNA topology, a popular target for cancer treatment. The α-isoform of topoisomerase II (topo II) is found in tumors at non-physiological levels, whereas the β-isoform is found mainly in proliferating and postmitotic cells. This variance makes the human topo IIα a prime target for cancer therapies in humans [23,24,25,26]. Topo II inhibitors are classically divided into topo II poisons and catalytic inhibitors, according to their mechanism of action. The former transform topoisomerases into cellular toxins which act by stabilizing the normally transient covalent topo II–DNA complex, leading to permanent breaks in DNA and, consequently, apoptosis [27]. Topo poisons have a storied history in cancer chemotherapy and their ranks boast established drugs such as etoposide, doxorubicin, and mitoxantrone [27,28,29]. Unfortunately, their administration is linked to severe adverse effects, such as the induction of secondary malignancies and cardiotoxicity [30,31]. In addition, resistance to existing cancer therapies, including topo II poisons, is rising [32,33]. In the hope of solving these problems, other inhibitory mechanisms are being explored, which has led to the development of a second group—catalytic inhibitors [30,34,35]. Such compounds act via four different mechanisms: interfering with DNA binding, DNA cleavage or ATP hydrolysis, or by binding to the ATP binding site [27,36]. 

In our previous studies, we have developed drug-like small catalytic inhibitors that target the ATP binding site of topo IIα and mimic the ATP adenine moiety with a variety of scaffolds, all of synthetic origin [37,38,39,40]. This time we explored another region of chemical space—compounds isolated from nature. While less ideal in terms of their crude properties and ability to function as immediate drug candidates, natural products can form novel molecular patterns and describe a chemical space that is rarely available in conventional synthetic libraries. As well, there is always molecular optimization to help enhance their initial attributes [41,42].

**Figure 1:**
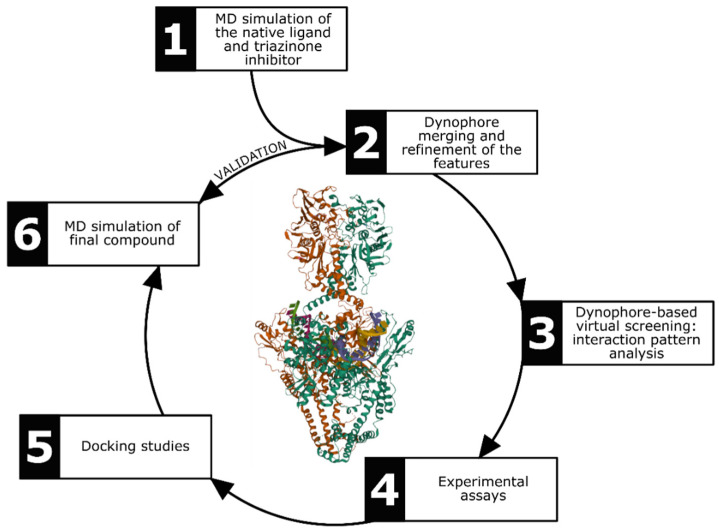
Outline of the research workflow based on dynophore models using human topo IIα as a model target.

In this study, we sought to deviate from classical screening protocols and incorporate the dynamic properties of our selected anti-cancer target. We developed a molecular design strategy (Figure 1) in which we first examined the dynamic behavior of protein–ligand complexes and constructed dynophore models for a native ligand analog and a known catalytic inhibitor. We then used the resulting data to first create a common master pharmacophore and then derived three sub-pharmacophores, i.e., static filters for rapid candidate selection. The identified hits were subsequently examined using various biochemical and biophysical methods in conjunction with in silico molecular simulations. Thus, we fully validated both our whole filtering protocol and our initial molecular recognition models.

## 2. Results and Discussion

### 2.1. Dynophore-Based Molecular Design Strategy

With the proposed design strategy, we aimed to incorporate a dynamic component of molecular recognition of known ligands that bind to the ATP-binding site of topo IIα into a structure-based pharmacophore that can be used in virtual screening. To achieve this goal, we integrated information from molecular dynamics simulations of protein–ligand complexes into our pharmacophore design pipeline (Figure 1). 

**Figure 2:**
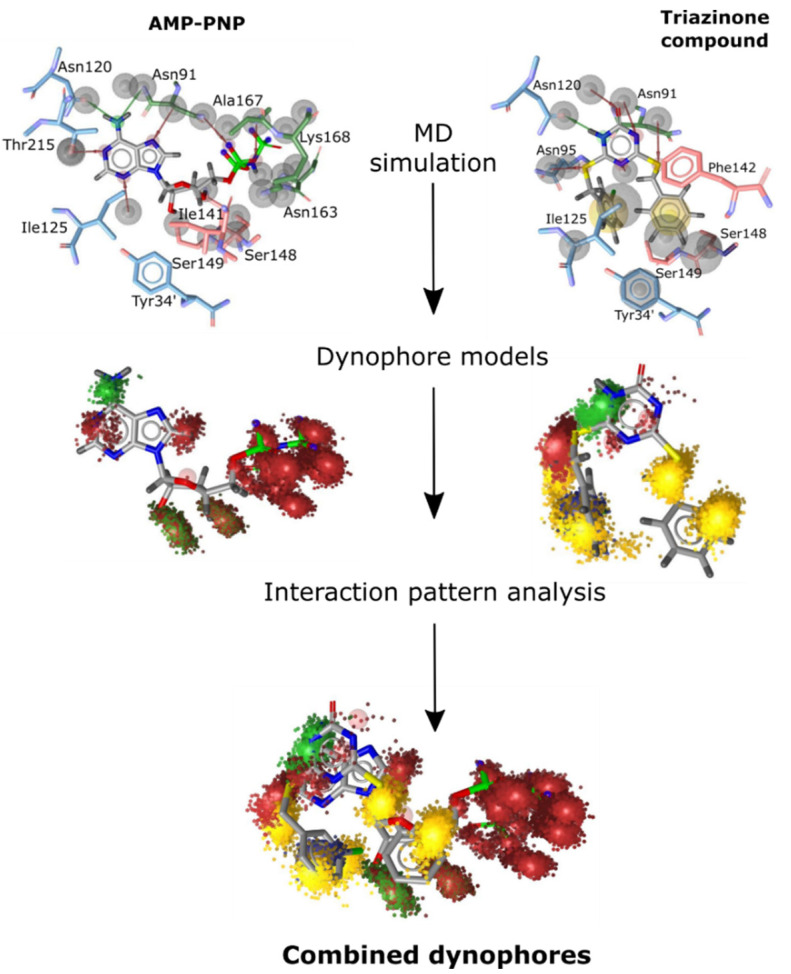
Dynamic Pharmacophores–Dynophores. After performing MD simulations of the AMP-PNP molecule and the catalytic triazinone inhibitor in the human topo IIα ATP site, the pharmacophore interactions were calculated over the course of each simulation, resulting in dynophore models. The obtained dynophore models were then analyzed and combined.

The design process of such a 3D structure-based pharmacophore model began with a comprehensive reanalysis of two previously performed 30 ns molecular dynamics simulations. One of the topo-IIα-ATPase in complex with a co-crystallized AMP-PNP molecule (a non-hydrolysable ATP analog), and the other in complex with a validated triazinone-class catalytic inhibitor designed to bind to the ATP site [37]. To better understand and accurately assess the interaction patterns and spatial distribution of the interactions of both ligands, we derived two dynophore models (dynamic pharmacophores) using DynophoreApp software (Figure 2). A dynamic pharmacophore allows us to combine the features of both MD simulations and static pharmacophore models, as it provides us with the statistical characterization of the pharmacophore features of the ligand throughout the simulation [18,43]. This information is then distilled into so-called “super-features” (Figure 2).

With dynophores, we could point out the key residues involved in the interactions that persist through most of the simulation time. Already, from the previously designed inhibitors [37,44,45,46], we recognized Asn120 as the key amino acid residue, exhibiting an anchor-like function via hydrogen bonding. Additional intermolecular interactions in the adenine portion of the binding pocket were found with the amino acid residues Asn91, Asn95, and Thr215; lipophilic interactions with the hydrophobic pocket enclosed by Ile125, Ile141, and Phe142; H-bond interactions in the sugar portion of the pocket with residues Ser148, Ser149, and Asn150. In the MD simulation of the native AMP-PNP, we also observed interactions in the phosphate portion of the binding site with residues Gly166, Tyr165, Lys378, and Arg162; however, these interactions were not present in the triazinone simulation due to its structure (Figure 2).

To filter out some of the less important interactions and to specify the interactions that are critical to the active site ligand recognition process, we next refined the dynophore features. We aligned the compounds and joined the two dynophores together. We then analyzed the aligned density clouds representing the pharmacophore features as shown graphically in Figure 2. The refinement criterion was based on the consistency of the interactions throughout the simulation and their significance observed in our previous studies at the same binding site. Our goal was to thoroughly investigate the ATP binding pocket. Therefore, we wanted to derive a balanced pharmacophore model that was broad enough to capture diverse compounds, but still restrictive enough to ensure specificity.

**Figure 3:**
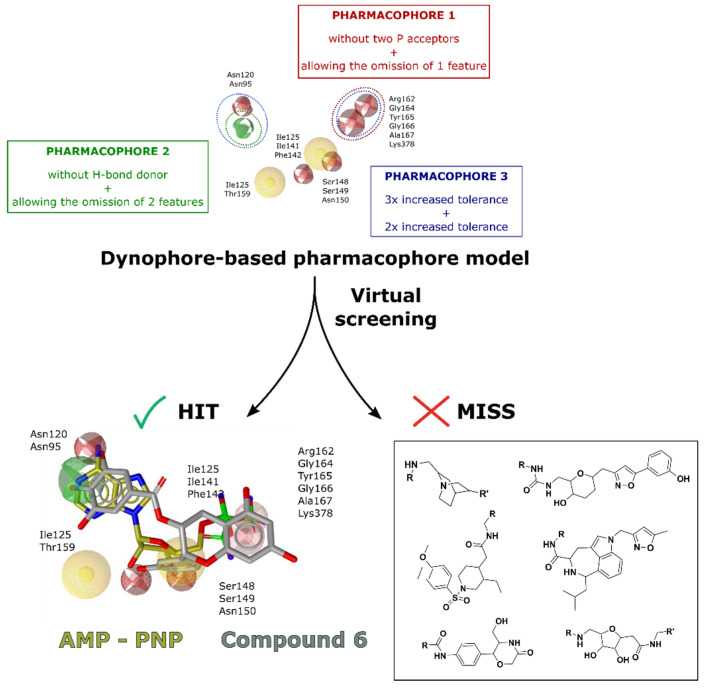
Dynophore refinement process in the creation of a new screening pharmacophore model. (**Top**) Designed Pharmacophore models 1–3 were used for virtual screening of compounds. Red spheres represent H-bond acceptors, green spheres represent H-bond donors, yellow spheres represent hydrophobic interactions. (**Bottom left**) An active flavonoid (compound **6**) aligned to Pharmacophore model 1 along with the experimental conformation of AMP-PNP for better comparison. (**Bottom right**) Structures of selected hit compounds that were found to be inactive in the experimental inhibition assay.

Based on the intermolecular interactions observed in the dynophore models, we retained the following features in the derived pharmacophore model: (1) the H-bonding features reflecting the interactions between the adenine ring or its mimetic in the triazinone scaffold and the amino acid residues Asn120 and Asn95 (present in both simulations), (2) the hydrophobic contacts observed in the triazinone compound simulation—those reflecting the interactions between the meta-chlorophenyl substituent, Ile125, and the hydrophobic pocket residues—Ile141, Phe142, and Thr159, (3) H-bond acceptor features observed in the MD simulation of AMP-PNP between the sugar moiety and residues Ser148, Ser149, and Asn150, and (4) H-bond acceptor features to capture the interactions of the AMP-PNP phosphate chain with the binding pocket. The resulting pharmacophore model with selected pharmacophore features is shown in Figure 3.

Our new pharmacophore model contains all important pharmacophore features to assure interactions which is in-line with our previously designed inhibitors targeting this site [37,38,39,40]. However, the number of features present would be too large and, consequently, too limiting to reasonably perform the virtual screening step. Therefore, we developed three submodels (Pharmacophores 1–3), each relaxing the constraints on a different part of the ATP binding pocket and exhibiting a different degree of relaxation (Figure S3).

In the first model, Pharmacophore 1, we omitted the phosphate acceptor region and allowed the omission of one feature. With this modification, we sought to identify more hit compounds that have structural elements which can mimic ATP interaction with the adenine and ribose pocket regions and also contain a lipophilic moiety that reflects the triazinone inhibitor substituent that lies outside the binding pocket. In Pharmacophore 2, we removed the H-bond donor criterion and allowed two features to be omitted during screening. This further simplification with a less restrictive feature of the ATP–Asn120 interaction and a lower number of required matched features for a hit molecule was planned to afford a more diverse section of hits from a broader chemical space. Finally, in Pharmacophore 3, we retained all pharmacophore features but fine-tuned their tolerance threshold. We increased the tolerance of the features in the adenine portion of the pocket by a factor of three and in the phosphate portion by a factor of two (Figure S3). Using LigandScout, we then performed three virtual screening campaigns with approximately 32,000 natural and semisynthetic compounds from the available AnalytiCon libraries, which were converted to a multiconformal format for screening. Each screening used one of the derived submodels, Pharmacophores 1–3, and a compound was considered a hit if it passed at least 1 screening.

Virtual screening yielded 32, 23, and 9 hits for Pharmacophores 1–3, respectively. An example of a hit molecule from Pharmacophore 1 is compound **6**, a natural flavonoid-based compound that showed good inhibition results in subsequent experimental assays. We visualized its identified conformation along with the conformation of the AMP-PMP molecule to compare its orientation and evaluate possible interactions in the ATP binding site (Figure 3). Compound **6** pairs well with the native ligand analog AMP-PNP in the adenine and sugar portions of the ATP binding site. However, the benzopyrene backbone of compound **6** does not overlap with the phosphates of the AMP-PNP molecule, which is to be expected since we omitted the P-acceptor region in Pharmacophore 1. In addition, compounds from several different chemical classes were also identified (Table S1 and Figure 3).

### 2.2. Human Topo IIα Inhibition Assays

To evaluate the utility of our screening approach, which took into account dynamic components of molecular recognition between a target and ligand; we first performed a High Throughput Screening (HTS) topo IIα relaxation assay. Experimental inhibition assays were performed for all hit compounds that were available, a total of 49. An initial topo IIα inhibition screen of compounds **1**–**49** was performed at concentrations of 20 µM and 200 µM, followed by titration at four different concentrations for the most promising compounds (see Table S1 for the structures of all tested compounds). Etoposide was used as a reference compound and its inhibition was comparable to the values reported in the literature [47].

Pharmacophore-based virtual screening identified several promising compound classes; however, ultimately only flavonoid compounds showed significant topo IIα inhibitory activity (Table 1). The identified active flavonoid compounds were **3**, **5**, **6**, and **7**, with **6**, epicatechin gallate isolated from green tea (*Camellia sinensis*), possessing the strongest IC_50_ inhibition value of 1.7 µM. The remaining flavonoid hits, **1**, **2**, **4**, and **8**, showed no topo II inhibition.

Following the HTS relaxation test, we examined the inhibitory mechanism of the agents in more detail. This is a necessary step in the evaluation of topo IIα inhibitors, as, due to the complex catalytic cycle, only additional assays can provide important information on how these compounds act at the molecular level [48]. In the case of flavonoids, it is particularly important to determine the inhibitory mechanism, since molecules of this class can act as either poisons or as catalytic inhibitors, and there are even conflicting reports for certain molecules [49,50,51,52].

We selected the two most potent compounds, **6** and **7**, for further study. First, a topo IIα-mediated decatenation assay was performed using etoposide as a control compound to validate the results of the HTS assay. This type of assay also allows direct for visual inspection of the inhibition process and is, in this aspect, superior to the HTS assay, in which inhibition is detected indirectly via spectroscopic detection of the formed triplex structure. In such assays, compounds that inherently exhibit strong fluorescence could yield misleading results.

**Figure 4:**
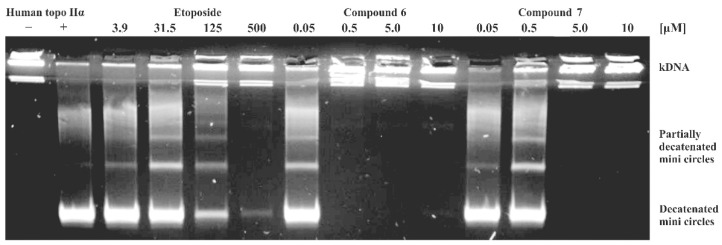
Results of the human topo IIα decatenation assay. The assay was performed at four different concentrations of compounds **6** and **7** (0.05, 0.5, 5.0, and 10 μM), and of etoposide as positive control (3.9, 31.5, 125, and 500 μM).

First, we analyzed the behavior of the etoposide control drug, where almost no topo II decatenation activity was observed in the presence of 125 μM and 500 μM compound concentration (Figure 4). Moderate enzyme activity was observed at the 31.5 μΜ, placing the IC_50_ value in the expected range between 31.5 μΜ and 125 μΜ [53]. Both flavonoids **6** and **7** inhibited the decatenation of kinetoplast DNA (kDNA), an aggregate of interlocked DNA minicircles, in a concentration-dependent manner and showed significantly higher inhibitory activity compared to etoposide. They displayed virtually complete inhibition of decatenation catalyzed by topo II at a concentration of 0.5 μM for compound **6** and 5 μM for compound **7** (Figure 4, with further details in Figure S4 and Table S2).

### 2.3. Investigation of the Inhibition Mechanism

**Figure 5:**
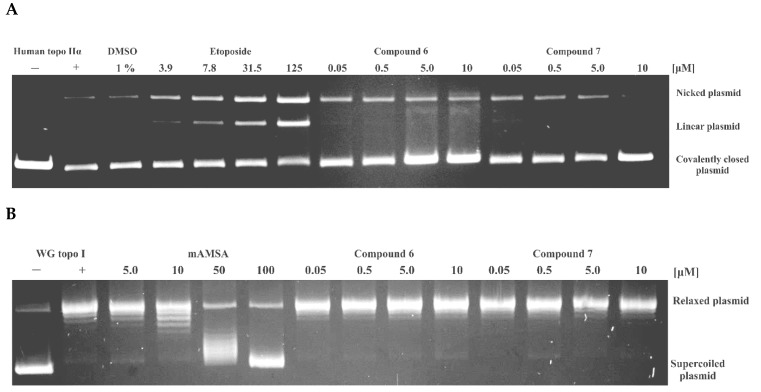
(**A**) Human topo IIα cleavage assay. The assay was performed at four different concentrations of compounds **6** and **7** (0.05, 0.5, 5.0, and 10 μM), and etoposide was used as a positive control (3.9, 7.8, 31.5, and 125 μM). (**B**) Unwinding assay. The assay was performed at four different concentrations of compounds 6 and 7 (0.05, 0.5, 5.0, and 10 μM), and of the positive control intercalator mAMSA (5.0, 10, 50, and 100 μM).

To determine whether our flavonoid compounds act as poisons or catalytic inhibitors, the cleavage assay was performed for compounds **6** and **7**, and etoposide was used as a control. The results are shown in Figure 5A (see Figure S5 and Table S3 for further data). Topoisomerase II poisons stabilize the usually transient covalent cleavage complex between the enzyme and DNA, and increase its concentration in a concentration-dependent manner. This results in an increase in the concentration of the linear (cleaved) plasmid when topo II is rapidly denaturated during the assay as the plasmid is prevented from being closed again. With gel electrophoresis, we can then distinguish between the different plasmid forms [48].

In the case of the topo II poison etoposide, higher amounts of linear plasmid were visible on the gel at higher concentrations. In the case of compounds **6** and **7**, significantly increased amounts of linear DNA were not observed at any of the concentrations tested, i.e., they acted as catalytic inhibitors, which is consistent with the mode of action expected based on our screening design. 

To further investigate whether compounds **6** and **7** act as intercalators, we performed the unwinding assay. The intercalating effects of the positive control, the intercalator mAMSA, on the supercoiled substrate could be observed, with complete intercalation/unwinding at 100 μM. Neither compounds **6** nor **7** showed any intercalating effects up to 10 μM (Figure 5B and Figure S6).

To investigate the effect on ATP hydrolysis, the ATPase assay was performed for compounds **6** and **7**. The percentage of average ATPase activity is shown in Table 2 (see also Table S4 and Figures S7 and S8). We chose higher concentrations for etoposide compared to compounds **6** and **7**, because its observed inhibitory effect was weaker. Compound **6** performed better than compound **7**, inhibiting ATPase activity at 10, 5, and 0.5 μM, with inhibition rates of 44.0, 39.7, and 31.8%, respectively. By comparison, etoposide inhibited ATPase activity at 250, 100, 50, and 25 μM, with inhibition rates of 82.6, 47.4, 30.3, and 20.1%, respectively. We also performed a competitive ATP assay, which showed a concentration-dependent effect of compound **6** on ATPase activity (Figure S10).

**Figure 6:**
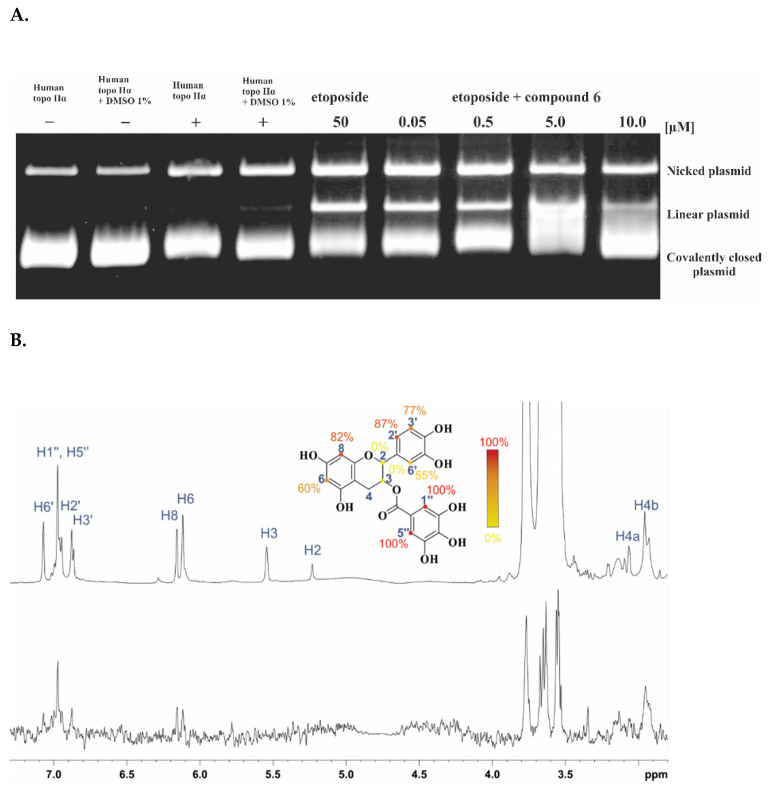
(**A**) Gel image of the first run of the topoisomerase IIα competitive cleavage assay. The assay was performed at 4 different concentrations of compound **6** (0.05, 0.5, 5.0, and 10 μM) in the presence of 50 μM etoposide. (**B**) 1D ^1^H STD NMR spectra for compound **6** recorded at topoisomerase IIα:ligand ratio of 1:100 and 600 MHz. The molecular structure illustrates the proton nomenclature and color-coded relative degrees of saturation of the individual protons. The STD amplification factors were normalized to the intensity of the signal with the largest STD effect. Reference STD spectra (above) with proton assignment and difference STD spectra (below) are shown. Water signals (4.63 ppm) were removed by the processing filter. H2 and H3 proton signals exhibit decreased intensity in the reference spectrum due to interference of water suppression applied at 4.63 ppm. H4a and H4b proton signals are overlapped with DTT proton signals; therefore, the relative STD amplification factors could not be determined. Proton signals between 3.5 and 3.8 ppm belong to the glycerol and are not shown in full size. Proton signals were calibrated to the DSS signal at 0.0 ppm.

A competitive cleavage assay was then performed to determine whether compound **6** could inhibit the formation of cleavage complexes induced by etoposide. The results showed that compound **6** reduced the linear product of etoposide at a concentration of 5.0 μM and above, i.e., it inhibited topo II poison activity (Figure 6A). The percentage of linear DNA in the presence of control etoposide alone was 19.8% and compound **6** reduced it to 14.2% at 5.0 μM and 5.6% at 10 μM (Figure S9 and Table S5).

We next examined the binding of flavonoid **6** to the isolated human topo IIα-ATPase domain, where the targeted ATP binding site is located. To this end, we expressed and purified the isolated ATPase domain of human DNA topoisomerase IIα (see Methods section). The interaction of compound **6** with the ATPase of topoisomerase IIα was successfully confirmed by the 1D ^1^H saturation transfer difference (STD) NMR experiment, suggesting a binding affinity (*K*_D_) of the compound in the micro- to millimolar range. STD signals were observed for all molecular moieties, and the relative values of the STD amplification factors showed the strongest interaction between the protons of the 3-OH-substituted phenyl ring and the protein (Figure 6B). In conclusion, the experiment indicated that the ATPase domain serves as the binding site of the identified natural product, compound **6**.

### 2.4. Computational Evaluation of Binding and Reconnection to the Initial Dynophore Model

The crystal structure of the human topo IIα ATPase domain in complex with a known small molecule inhibitor bound to the ATP binding site is unfortunately not yet available. In such cases, the first measure is usually to construct a static protein-ligand model using mostly rigid molecular docking. However, dynamic methods can provide us with even more valuable information [5]. Protein flexibility can be taken into account during the creation of the binding pose itself, e.g., by induced fit or ensemble docking, and/or at a later stage, e.g., by MD and trajectory analysis [54,55]. For the creation of the binding model and the MD simulations, we chose compound **6**—the compound with the highest inhibition potential, as suggested by the experimental assays.

**Figure 7:**
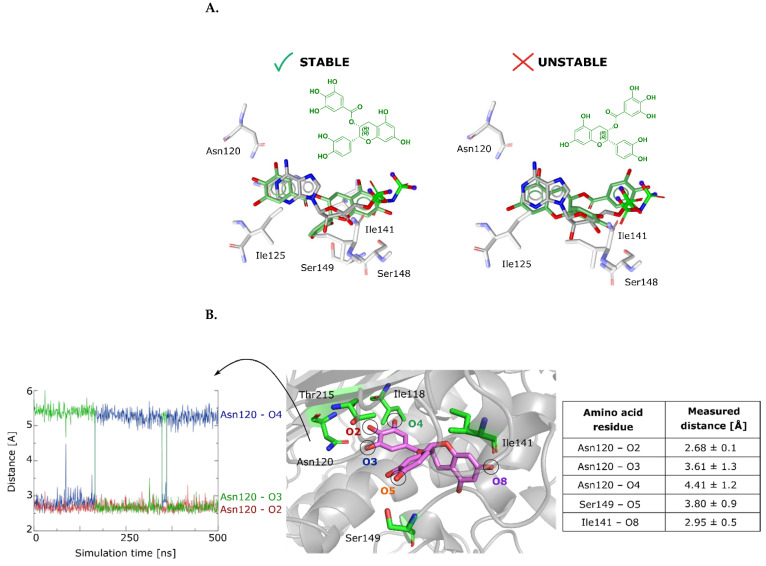

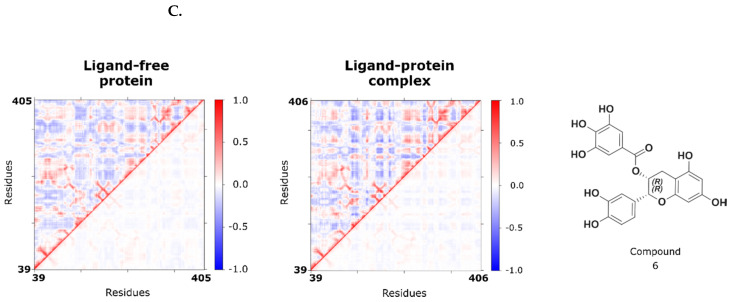
Molecular recognition of compound **6** and human topo IIα ATP binding site (PDB: 1ZXM). (**A**) Two alternative conformations of compound **6** in the ATP binding site identified by molecular docking. (**B**) Molecular simulation of the topo IIα: compound **6** complex. (**Right**) A snapshot from the MD simulation showing measured distances between compound **6** and surrounding residues. (**Left**) A complex interplay of OH groups that can interact interchangeably with Asn120. (**C**) Calculated cross-correlation matrices of the ligand-free and ligand 6-bound topo IIα systems. Residue 406 designation in the second matrix marks compound **6**.

Compound **6** isolated from green tea *Camellia sinensis* stereochemistry was determined as (*R,R*), and we took this configuration as the starting point for molecular docking. The validated molecular docking protocol revealed two predominant orientations of **6**, one with interactions consistent with the Pharmacophore 1 model and an alternative one mirrored horizontally (Figure 7A).

We then ran two 0.5 µs MD simulations, one for each orientation. Without even performing a detailed analysis of the obtained MD trajectories, we could already visually observe that the simulation of the alternative conformations was very unstable—the ligand did not stabilize in the binding pocket. Therefore, we discarded it and focused only on the complex in which ligand **6** was placed in agreement with the screening pharmacophore model. In the same way, we also performed the MD simulation of a protomer of the N-terminal domain of topo IIα without any ligands to observe and analyze the differences between the domains in terms of amino acid residue movement.

At this point, we should also note that, given the considerable differences in the substituents of the four active flavonoids and the distribution of their OH groups, as well as the tendency of flavonoids to show multiple orientations, the other three compounds could be oriented differently. For example, in the case of compound **7**, the docking calculations revealed only the alternative pose. The network of OH groups, which can be strategically placed at different positions in the flavonoid molecules, can enter into different hydrogen bonding networks, making the binding highly unpredictable [56]. This circumstance might explain the different modes of action of flavonoids observed on the topo II enzyme (Table S7) [49,50,51,52]. While attempts have been made to establish guidelines for which substituents might lead to a particular topo II inhibition mechanism, some studies have even observed different inhibitory mechanisms for the same compound (e.g., the flavonoid quercetin has been described as both a catalytic inhibitor and a topo II poison). This could indicate that intracellular conditions such as pH might influence the binding mechanism of flavonoids.

We began our analysis of the MD trajectory by calculating commonly used geometric parameters and analyzing some distances between ligand **6** and the surrounding amino acids to quickly look for potentially important interactions. The root-mean square deviation (RMSD) value of the protein calculated for all Cα atoms shows that the deviation from the initial structure is reasonable (2.61 ± 0.3 Å), which means that it was stable throughout the simulation time. The predominant source of structural deviation can be attributed to the movement of the DNA gate. The Root-Mean Square Fluctuations (RMSF) calculation revealed that the highest fluctuations were associated with the transducer domain region of the protein (residues 265–405), and that the area encompassing the ATP site was stable. It seems that this flexibility of this region does not affect the binding of the ligand. The ligand was also stable overall; the RMSD was 1.62 ± 0.2 Å (Figure S12). However, examination of the trajectory reveals an interesting shift at about the 150 ns mark. At the beginning of the MD simulation, the ligand has not yet fully settled at its binding site, leading to fluctuations in the first 150 ns. Thereafter, the trihydroxybenzene portion of the molecule shifts toward Asn120 and the H-bonding interactions of the hydroxyl groups change as follows: the O2 shifts away from Asn95 but maintains its bond with Asn120, O4 shifts from Ile118 to Asn120, and O3 shifts from Asn120 and Thr215 to Ile118 (Figure 7B and Figure S13). In all cases, the groups act as H-bond donors. In essence, the ligand has adjusted its position to resemble the original screening pharmacophore model even more closely.

Next, we measured some of the key distances between compound **6** and the amino acid environment. Upon visual inspection of the trajectories, we found some residues that have the potential to form hydrogen bonds with our ligand. However, after measuring the average distances, it became apparent that many potential interactions had higher average distances than expected. This is a consequence of our ligand containing many closely spaced hydroxyl groups on a branched scaffold, which allows for a multitude of interactions. Consequently, even slight conformational shifts of the ligand’s side moieties result in notable changes in the interaction pattern. An example of such behavior can be seen in Figure 7B (left), where the distal phenyl substituent with three OH groups (O2–O4) moves slightly, causing O3 and O4 to alternately form interactions with Asn120.

In Figure 7B, we show the distances of Asn120, Ser149, and Ile141, which were the most stable during MD. The typical bond length for hydrogen bonds is between 2.2 and 3.8 Å [56], thus, the observed amino acids show good potential to form H-bonds.

To observe whether the presence of the ligand affects the movements of the protein residues, we calculated cross-correlation matrices consisting of normalized cross-correlation coefficients on a scale between −1 and +1. As can be seen in Figure 7C, the cross-correlation matrix of the ligand-free protein shows minimal movements of the protein and its residues. In contrast, the matrix of the ligand-protein complex shows more definite anti-correlation movements. We also visualized and compared the anti-correlation movements for the topo IIα complex with bound flavonoid **6** versus the ligand-free structure (Figure S14). The complex exhibits a stronger anti-correlation movement (maximum anti-correlation coefficients ranging from −0.4 to −0.6) and is concentrated around the ATP binding site and the DNA gate. On the other hand, the ligand-free system shows smaller anti-correlation movements (maximum coefficient values −0.2 to −0.4), which are distributed throughout the protein without any discernible pattern. This suggests that while the ligand-free protein is in a passive state, the binding of compound **6** results in an enhanced and focused movement. This could be due to the fact that our ligand mimics some of the interactions of the native ligand, as also corroborated by the STD NMR binding experiments.

As we have seen in our case, a simple geometric approach is not sufficient to study the interaction pattern. However, H-bonds are not the only type of interaction that cannot be fully captured by geometric analyses, another example being hydrophobic contacts. These types of interactions can be observed well with static and dynamic pharmacophore models. Thus, we again utilized the dynophore model approach to analyze the simulation data.

**Figure 8:**
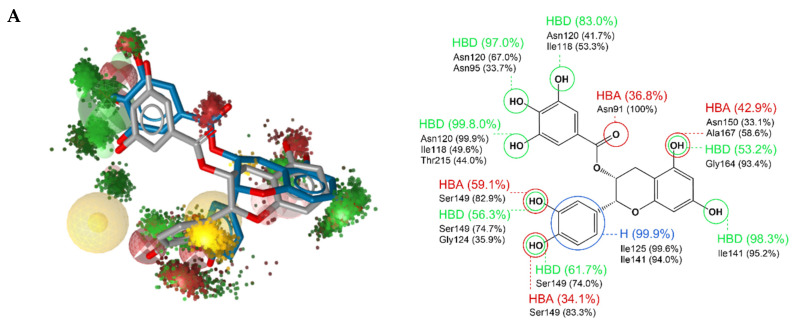

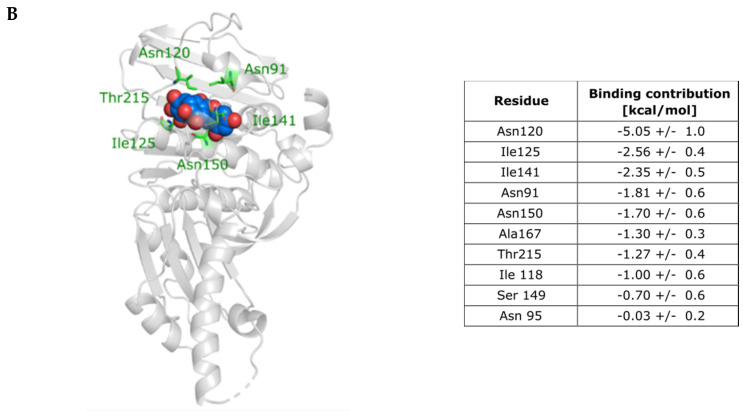
(**A**) Dynophore model for the selected compound **6**. (**Left**) Overlay of our 3D dynophore model with the point cloud representation of superfeatures with the screening pharmacophore and overlay of the conformation of compound **6** from pharmacophore screening (grey) with the conformation of compound **6** from the later stages of the MD simulation (blue). (**Right**) Detailed insights into the interaction patterns with amino acid residues and statistical information). (**B**) Per-residue energy decomposition of binding free energy. Compound **6** is depicted in blue and the residues that contribute the most to its binding are in green.

The dynophore model we obtained for our ligand is shown in Figure 8A and provided as an animation in Supporting Information. On the left side of Figure 8A, we also overlaid the dynophore with our screening pharmacophore. The dynophore is represented graphically with superfeature clouds and, on the right-hand side, these superfeatures are broken down into the contributions of each residue to each interaction. We can observe a high proportion of hydrogen bonding and a hydrophobic interaction between the ATP binding site and the ligand. 

As for the specific amino acid residues involved in the interactions, we can see that they are consistent with the designed interaction pattern from the virtual screening Pharmacophore l. We see that residue Asn120 is indeed strongly involved in hydrogen bonding and interacts with one of the three OH groups of the phenyl ring, together with minor contributions from residues Ile118, Thr215, and Asn95. It was gratifying to observe that the prevalence of the interactions between the protein and the trihydroxybenzene group observed with STD-NMR (Figure 6B) was also reflected here. On the second phenyl ring, we observe hydrogen interactions with residue Ser149, and a strong hydrophobic interaction formed with Ile125 and Ile141. 

There are also additional interactions that were not part of the screening Pharmacophore 1. For example, the hydrogen bond acceptor interaction with Asn91. Furthermore, there are also hydrogen bond acceptor and donor interactions in the phosphate tail portion of the binding pocket, where the main scaffold forms interactions via the OH groups of the phenyl ring with amino acid residues Gly164, Asn150, Ala167, and Ile141. These interactions partially overlap with one of the H-bond acceptor spheres from the master screening pharmacophore, which was eliminated for Pharmacophore 1 to avoid too much restriction. It should be noted that there are several regions in the dynophore where H-bond acceptor and donor superfeatures overlap, which is due to the fact that the OH groups can play the role of both donors and acceptors and the binding pocket provides sufficient space. In our particular case, this means that the features describing the sugar and phosphate binding part of the ATP binding pocket should probably be considered as both donors and acceptors in future screenings. More broadly, one should consider whether the binding pocket can accommodate both a donor and an acceptor in the same location before committing to a single feature.

Finally, to also gain insight into the energetic aspect of molecular recognition, we evaluated the binding thermodynamics of compound **6** by performing MM/GBSA (Molecular Mechanics/Generalized Born Surface Area) endpoint free energy calculations. The estimated binding free energy ∆G_bind was −35.13 ± 3.7 kcal/mol, indicating a ligand with high affinity able to form a stable complex. 

From the point of view of rational drug design, it is important to understand which are the crucial residues for drug-target interactions; therefore, we also calculated the energy decomposition per residue. The results are presented in Figure 8B. Unsurprisingly, the highest energy contribution comes from Asn120, followed by smaller contributions from other residues already considered important for binding, e.g., Ile125 and Ile141. 

The energy decomposition is mostly consistent with the results of the geometry-based dynophore model, and both exclude some residues that were only highlighted during the docking process, i.e., Lys123 and Lys157. These two amino acids were also not part of the original pharmacophore-based screening boundary conditions. Nevertheless, some discrepancies remain. The interaction with Gly164 was found to be important in the dynophore; however, the energetic analysis assigns little importance to it. This indicates the complexity of molecular recognition and the need for experimental evaluation through structural studies that can provide final insight. 

Finally, we selected a small subset of 10 flavonoids with determined topo II activities (Table S7) and screened it against all three utilized Pharmacophores 1–3. The screening, unsurprisingly, returned zero hits. Developed pharmacophores 1–3 were intended to fully explore the large topo IIα ATP binding size, thus being intrinsically biased towards larger molecules, i.e., flavonoids with bigger substituents, and most of the flavonoids of the evaluated subset contain smaller substituents. Interestingly, even larger flavonoids in the set, such an epigallocatechin gallate (EGCG), were not identified as hits, despite being structurally similar to the active compound **6**. It is well known that general in silico screening methods tend to identify a small subset of representatives from each chemical class; obtaining a hit is but the crucial first step and, to fully define the SAR, one then needs to employ methods focused on highlighting derivates. In our case, the performed dynophore-based screening campaign allowed us to fish out an as-of-yet uncharacterized subset of flavonoids with molecular properties adhering to the designed screening pharmacophores.

## 3. Conclusions

The molecular design of biologically active compounds is a multifaceted, constantly evolving process. As the number of tools and methods continues to grow, it has become abundantly clear that the dynamic properties of protein targets should not be overlooked. Here, we present a molecular design strategy that incorporates protein flexibility at a modest computational cost and demonstrate its effectiveness using a well-known anti-cancer target protein—human DNA topoisomerase IIα. Based on MD simulations of a known catalytic inhibitor and an analog of the native ATP ligand, we derived a joint dynophore model that served as the basis for subsequent pharmacophore models that we used to screen chemical libraries. Experimental evaluation of the hits confirmed that some of the flavonoid compounds act as catalytic inhibitors of topo IIα, and they suggested interaction with the ATPase domain. The binding mode of the selected hit compound at the ATP target site was investigated in extensive molecular simulations and analyzed both geometrically and energetically. The new computational results agreed well with both our initial in silico hypothesis and the in vitro behavior of the hit compounds, thus validating the original dynophore-based screening model. A possible further step in the development of our method could be to also consider the binding energies of the original ligands in the construction of the screening pharmacophores. In summary, we showcased a new screening strategy that incorporates dynamic components of molecular recognition by expanding the known chemical space of inhibitors of topo IIα.

## 4. Materials and Methods

### 4.1. Generation of Dynophore Models and Pharmacophore-Based Virtual Screening

Initial dynophore models were generated using the DynophoreApp developed in the Molecular Design Lab at Freie Universität Berlin [57]. One thousand equidistant frames of the previously simulated AMP-PNP ligand [58] and our catalytic topo IIα triazinone-based inhibitor in the ATP binding site were used for each dynophore model generation [37]. These calculations were performed at FU computers and subsequently analyzed and visualized in LigandScout [59]. 

For virtual screening, we used AnalytiCon MEGx, NATx, and MACROx libraries of natural and semisynthetic compounds. The total number of compounds screened was approximately 32,000, and 3D conformational libraries of all molecules were created with the LigandScout Conformer Generator using the iCon Best setting. The subsequent virtual screenings were performed using LigandScout, taking exclusion volumes into account, and the obtained hits were scored using its pharmacophore fit scoring function. 

### 4.2. Human Topoisomerase IIα HTS Inhibition Assay

The assay was performed using the Human Topo II alpha Relaxation Assay Kit from Inspiralis (Norwich, UK). After rehydration of the wells using Wash buffer (20 mM Tris–HCl (pH = 7.6), 137 mM NaCl, 0.005% (*w*/*v*) BSA, 0.05% (*v*/*v*) TWEEN-20^®^), biotinylated oligonucleotide was immobilized in the wells. The excess oligonucleotide was washed off with Wash buffer. 

Next, the enzyme was incubated with the substrate (supercoiled plasmid pNO1) in a reaction volume of 30 μL. Enzyme was diluted to appropriate concentration with dilution buffer (50 mM Tris–HCl (pH = 7.5), 100 mM NaCl, 1 mM DTT, 0.5 mM EDTA, 50% (*v*/*v*) glycerol, 50 μg/mL albumin). Then, 3 μL of tested compounds, diluted in 10% DMSO, were added into the well, except for positive and negative control. The final concentration of DMSO was 1%. Mixtures were incubated at 37 °C for 30 min, then TF buffer (50 mM NaOAc (pH = 5.0), 50 mM NaCl, 50 mM MgCl_2_) was added to the wells and incubated at room temperature for an additional 30 min to allow triplex formation. With the aim of eliminating the aggregation and non-specific inhibition, a surfactant (0.008% TWEEN-20^®^) was added in the reaction mixture. Unbound plasmid was washed off with TF buffer and stained with DNA-detection Dye in T10 buffer (10 mM Tris–HCl (pH = 8) and 1 mM EDTA). After mixing, fluorescence was read using Synergy Mx (Biotek) (Excitation: 495 nm and Emission: 535 nm). Etoposide was used as a positive control. Applying this protocol, we first screened all selected hit compounds **1**–**49** at compound concentrations of 20 μM and 200 μM (Table S1). For all hit compounds from the flavonoid class, a titration assay was performed; at concentrations of 2, 50, 100, and 200 μM for compounds **1**, **2**, **3**, **4**, **5**, **8**, and etoposide standard, and at 0.05, 0.5, 5.0 and 50 μM concentrations for compounds **6** and **7**. The IC_50_ values were calculated with GraphPad Prism 7.0 [60]. All screening experiments were performed in duplicates.

### 4.3. Human Topoisomerase IIα Decatenation Assay

This, and the assays described in Section 4.4, Section 4.5 and Section 4.6, were performed in collaboration with Inspiralis (Norwich, UK). One U of topo II was incubated with 200 ng kDNA in a 30 μL reaction at 37 °C for 30 min under the following conditions: 50 mM Tris HCl (pH 7.5), 125 mM NaCl, 10 mM MgCl_2_, 5 mM DTT, 0.5 mM EDTA, 0.1 mg/mL bovine serum albumin (BSA), and 1 mM ATP. The reaction was then stopped by the addition of 30 μL chloroform/iso-amyl alcohol (26:1) and 30 μL Stop Dye (40% sucrose (*w*/*v*), 100 mM Tris. HCl (pH 7.5), 10 mM EDTA, 0.5 μg/mL bromophenol blue) before being loaded on a 1% TAE gel run at 85 V for 90 min. 

Bands were visualized by the ethidium bromide staining for 15 min and destaining for 10 min. Gels were scanned using documentation equipment (GeneGenius, Syngene, Cambridge, UK) and inhibition levels were calculated from the band data obtained with the gel scanning software (GeneTools, Syngene, Cambridge, UK). Assay was performed for active compounds **6** and **7** at concentrations of 0.05, 0.5, 5.0, and 10 μM, and for etoposide standard at concentrations 3.9, 31.5, 125 and 500 μM.

### 4.4. Human Topo IIα Cleavage and Competitive assay

One U of the human topo IIα was incubated with 0.5 μg supercoiled plasmid DNA (pBR322) in a 30 μL reaction at 37 °C for 30 min under the following conditions: 20 mM Tris HCl (pH 7.5), 200 mM NaCl, 0.25 mM EDTA, and 5% glycerol. The reaction was then incubated for a further 30 min with 0.2% SDS and 0.5 μg/μL proteinase K. The reaction was then stopped by the addition of 30 μL chloroform/iso-amyl alcohol (26:1) and 30 μL Stop Dye (40% sucrose (*w*/*v*), 100 mM Tris HCl (pH 7.5), 10 mM EDTA, 0.5 μg/mL bromophenol blue) before being loaded on a 1% TAE gel run at 80 V for 2 h. Bands were visualized as described in the decatenation assay. Assay was performed for compounds **6** and **7** at concentrations of 0.05, 0.5, 5.0, and 10 μM and for etoposide (control) at concentrations of 3.9, 7.8, 31.5, and 125 μM.

The competitive cleavage assay was performed as described above at the same 4 concentrations of compound **6**, with the difference being that a constant etoposide concentration of 50 μM was also present. The amount of etoposide required for optimal cleavage was determined by prior titration. Total DMSO in the assay was 2% (cumulative effect of adding 2 different compounds). Cleavage and competitive cleavage assays were performed in duplicate for all compounds.

### 4.5. Wheatgerm Topo I Unwinding Assay

Compounds **6** and **7** were incubated with an excess of wheatgerm topo I and 0.5 μg supercoiled plasmid DNA in a 30 μL reaction at 37 °C for 30 min under the following conditions: 50 mM Tris-HCl (pH 7.9), 1 mM EDTA, 1 mM DTT, 20% (*v*/*v*) glycerol, and 50 mM NaCl. Compounds were diluted from 50 mM stocks in 100% DMSO; 0.3 μL of each compound was added to assay. Final DMSO concentration in the assay was 1%. Final compound concentrations were: 0.05, 0.5, 5.0, and 10 μM; 20 μL of water and 50 μL of butanol (water-saturated) were then added to extract the compounds and the reaction was then stopped by the addition of 30 μL chloroform/iso-amyl alcohol (26:1) and 30 μL Stop Dye (40% sucrose (*w*/*v*), 100 mM Tris-HCl (pH 7.5), 10 mM EDTA, 0.5 μg/mL bromophenol blue) before being loaded on a 1% TAE gel run at 80 V for 2 h. Bands were visualized as in the decatenation assay.

### 4.6. ATPase Assay of Human Topo Iiα

ATPase assay of the human topoisomerase IIα was performed using a pyruvate kinase/lactate dehydrogenase assay, which measures the reduction of NADH at 340 nm. Conversion of NADH to NAD is caused by ADP, which is formed from the ATP hydrolysis. A mixture of linear pBR322 (1.5 μL of 1 mg/mL per assay), assay buffer (20 mM Tris−HCl, 125 mM potassium acetate, 5 mM magnesium acetate, 2 mM DTT, pH 7.9), phosphoenol pyruvate (0.5 μL of 80 mM per assay), pyruvate kinase/lactate dehydrogenase mix (0.75 μL per assay), NADH (1 μL of 20 mM per assay), and water (34.35 μL per assay) was prepared. The prepared mixture (41.1 μL) was put into the wells on a 384-well microtiter plate. DMSO (0.5 μL), etoposide, or compound **6** or **7** were added to the wells and mixed. The dilution buffer (5 μL) or human topo IIα (12 nM final concentration) was then added and mixed. Then, a measurement of OD_340_ change was performed in a plate reader over a 10 min timeframe (called the prerun). Then, 3.4 μL of 30 mM ATP was added and the OD_340_ was monitored for the next 30 min. The assay temperature was 37 °C. The final DMSO concentration in all the reactions was 1% (*v*/*v*). Assays were performed in duplicates at 0.05, 0.5, 5, and 10 μM final concentrations of the investigated compounds **6** and **7**, and etoposide as the control compound at 25, 50, 100, and 250 μM.

### 4.7. Expression and Purification of ATPase Domain of Human Topoisomerase IIα

Expression and purification of the ATPase domain of human topoisomerase IIα followed an established protocol with some modifications [61]. Cultures of *S. cerevisiae* carrying the pYEPG plasmid for ATPase domain expression (generous gift from the late Prof. Tao-Shih Hsieh, Duke University Medical Centre, Durham, NC, USA) were grown on SD-U agar plates (Sintetic Dropout media *w*/*o* uracil (Sigma, St. Louis, MO, USA), YNB *w*/*o* AA (Sigma), (NH_4_)_2_SO_4_, 2% glucose, agar) at 30 °C for 48 h. A single colony was transferred into 5 mL SD-U medium and incubated overnight at 30 °C and 180 rpm. Then, 2 mL of the overnight culture was transferred into 160 mL SD-U medium and incubated overnight at 30 °C and 180 rpm. Finally, the overnight culture was used to inoculate 4 L YPGal medium (yeast extract, peptone, 2% galactose, YNB *w*/*o* AA, (NH_4_)_2_SO_4_) and incubated at 30 °C for 30 h at 180 rpm. Cells were harvested by centrifugation. The pellet was washed with 200 mL distilled water and centrifuged again. Pelleted cells were frozen and stored at −80 °C until use.

Cells were lysed in sorbitol buffer (1 M sorbitol, 25 mM K phosphate, 10 mM MgCl_2_, 20 mM BME) with zymolyase (R) 20T (Amsbio) by incubation at 30 °C. After 30 min, the suspension was centrifuged and the pellet was resuspended in imidazole buffer (15 mM imidazol, 10 mM MgCl_2_, 0.2 % Triton-X-100, 2 mM PMSF, 2 µg/mL leupeptin, 2 µg/mL pepstatin, 5 mM BME). After the homogenization using Dounce homogenizer, NaCl was added to the final 0.5 M and the suspension was centrifuged. The His-tagged protein was purified from the filtered supernatant on a 10 mL Ni-NTA Superflow column (GE Healthcare, Chicago, IL, USA), equilibrated in 15 mM imidazol, 10 mM MgCl_2_, 500 mM NaCl, and pH 7.0. To wash the impurities, the imidazole concentration was increased to 80 mM. Protein was eluted from the column with 400 mM imidazole and further purified with size-exclusion chromatography using 120 mL Superdex 75 PG column (GE Healthcare), which was equilibrated in 50 mM Tris-HCl, 100 mM NaCl, 0.5 mM EDTA, pH 7.4, and 20 mM BME. The purity and molecular weight of the eluted protein were determined using SDS-PAGE. The protein was then dialyzed against the storage buffer (50 mM TRIS HCl, 100 mM NaCl, 0.5 mM EDTA, 50% glycerol, 1 mM DTT, pH 7.4).

### 4.8. STD NMR Spectroscopy Experiments

The 1D STD ligand epitope mapping experiment [62] was recorded on a Bruker Avance Neo 600 MHz spectrometer using a cryoprobe at 25 °C. Data were collected using the pulse sequence provided by the Bruker library of pulse programs and analyzed with Bruker Topspin 4.0.7. The residual water signal was suppressed using excitation sculpting [63]. A T_1ρ_ filter of 30 ms was used to eliminate the background protein resonance. The ^1^H spectral width was 5263 Hz. The NMR sample was prepared in a buffer containing 20 mM K-phosphate (pH 7.7), 150 mM KCl, 5 mM MgCl_2_, 1 mM DTT (98%, D10), and 5% DMSO (99.9%, D6) in H_2_O. Spectra were recorded at a protein:ligand ratio of 1:100, where protein concentration was 2 µM and ligand concentration was 200 µM. The experiment was performed with 8192 data points, a relaxation delay of 2 s, and 8000 scans. Protein saturation time was 1.5 s, where selective saturation was achieved by a train of 50 ms long Gauss-shaped pulses separated by 1 ms delay. The on-resonance selective saturation of topoisomerase IIα was applied at −0.70 ppm. The off-resonance irradiation was applied at 30 ppm for the reference spectrum. Spectra were zero-filled and apodized by an exponential line-broadening function of 3 Hz.

### 4.9. Molecular Docking Calculations

Molecular docking experiments were performed using the GOLD software [64] and the human topo IIα ATPase domain (PDB: 1ZXM) [65]. In the first step, similarly to our previous studies [45,61], the validation of the GOLD tool was performed by redocking the co-crystalized AMP-PNP molecule into its binding site on chain A. The active site was defined as 10 Å radius around the co-crystallized ligand, and hydrogen atoms were added to the protein. Magnesium ions and all waters were removed except for W924 and W931. They were included in docking because previous studies suggest they play an important role in binding of the native molecule [46,66]. The AMP-PNP molecule was docked into the defined active site by applying the following parameters of the GOLD genetic algorithm (GA): population size = 100, selection pressure = 1.1, No. of operations = 100,000, No. of islands = 5, niche size = 2, migrate = 10, mutate = 95, and crossover = 95. Different spin states of the water molecules W924 and W931 were allowed during docking. As in previously published studies performed at with this structure [46], to ensure that interactions similar to the interaction pattern of the purine moiety in the co-crystallized AMP-PNP molecule would be obtained, an H-bond constraint was added to Asn120. The GoldScore scoring function was used. Obtained binding poses of docked AMP-PNP closely resembled the x-ray pose of AMP-PNP, with the best RMSD agreement of 0.9 Å, which indicated that the used docking setting are reliable (Figure S11). The same scoring function and the described docking settings were used for the molecular docking calculations of active flavonoid compounds that were identified in the pharmacophore-based virtual screening. Docking calculations were visualized and geometrically analyzed in LigandScout [59].

### 4.10. Molecular Dynamics Simulations

Molecular dynamics (MD) simulations were performed at the Azman high-performance computing (HPC) center at the National Institute of Chemistry in Ljubljana. For the simulation of the protein-ligand **6** complexes obtained via the above docking step, we started with the parameterization of ligand **6**. The partial charges of the ligand (Table S6) were obtained by performing a population analysis according to the Merz–Kollman scheme on the geometry optimized structure at the Hartree–Fock level using the 6–31 G* basis set. For the QM optimization, Gaussian 16 was used [67]. RESP charges were then generated with the Antechamber module of Amber18 [68]. The remaining ligand’s force field parameters were obtained with Antechamber module, using as input bond lengths and bond angles obtained from the optimized geometries. The General Amber Force Field of second generation (gaff2) was used for the ligand description [69]. 

The three to-be-simulated systems consisted of either the monomeric human topo IIα ATPase domain alone (chain A, residues 39–345, 350–405) or the monomer in complex with one of the two binding conformations of compound **6** in the ATP site. They were solvated using TIP3P type water molecules [70] in a cubic box with at least 10 Å from the solute to the edge of the box. Neutral charge of the system was achieved by adding 3 chloride ions. The system contained no surrounding ions to mimic the ion concentration under physiological conditions. Recent reports have shown that the influence of ionic strength on protein-ligand binding affinity is diverse and difficult to predict [71]; therefore, we opted to keep the simulation conditions consistent with our previous studies. The adequacy of the molecular simulation parameters was later corroborated with experimental binding data (see Section 2.4). 

The final systems contained approximately 113,000 atoms. Amber14SB force field was used for protein [72] and gaff2 for the ligand **6** [69]. Systems were submitted to an energy minimization of 10,000 steps applying steepest descent, followed by 20,000 steps of conjugate gradient optimization method. This was followed by an NVT equilibration (4 runs—each 10,000 steps with the time step of 2 fs, with gradually releasing constrain on the protein. Namely, force constant for the first run was 100 kcal mol^−1^ Å^−2^, second 60 kcal mol^−1^ Å^−2^, third 30 kcal mol^−1^ Å^−2^ and fourth was without restraint). This was followed by NPT equilibration (2 runs—100,000 steps with time steps of 2 fs); in the first run, the topo IIα ATPase domain was constrained with the force constant of 20 kcal mol^−1^ Å^−2^ and, in the second run, no constraint was applied. During the NVT equilibration, the systems were gently heated to reach a target temperature of 300 K, controlled by the Langevin thermostat. During the NPT equilibration, the pressure was maintained at 1 bar using the Berendsen barostat. Particle Mesh Ewald [73] has been used to treat long-range electrostatics, and periodic boundary conditions were applied. The SHAKE algorithm [74] was applied to constrain all bond lengths involving hydrogen atoms to achieve the time step of 2 fs. A total of 0.5 microsecond of simulation was then performed for each system. MD simulations were performed using the Amber18 code cuda program.

Trajectories obtained by the MD simulations were inspected and analyzed using the following tools. Cpptraj module of Ambertools 18 [75] was used to calculate the root mean square deviation (RMSD), the root mean square fluctuations (RMSF), interaction analysis, and distance measurements, and Bio3D [76] library (version 2.3-0) within the R environment [77] was used to calculate the dynamic cross-correlation maps (DCCM). Visualizations of the results were created using Visual Molecular Dynamics (VMD) software [78], R software environment [77], PyMOL [79], and UCSF Chimera [80]. Below, we provide some details of the performed analysis:

#### 4.10.1. Cα RMSD and RMSF Calculation

Cα RMSD and RMSF analyses were performed on the whole trajectory using the Cpptraj module. The RMSD and RMSF values were calculated referring to the initial structure of the protein and ligand, respectively.

#### 4.10.2. Interaction Analysis and Distance Measurements

Hydrogen bond analysis and distance measurements were performed on the converged part of the trajectories (100–500 ns), determined by RMSD and RMSF calculations. 

#### 4.10.3. Dynamical Cross-Correlation Map (DCCM) Analysis

The cross-correlation maps, determining the extend of pairwise residual correlations, were calculated with the Bio3D package, using the dccm function, which internally derives the covariation matrices and calculates the Pearson’s correlation coefficients ($C_{ij}$) on the Cα atom pairs *i* and *j* by applying the following equation:

$$C_{ij}=\frac{c_{ij}}{{\left[c_{ij}c_{ij}\right]}^{1/2}}=\frac{\langle \Delta r_{i}\;\cdot \;\Delta r_{j}\rangle }{\;\langle \Delta r_{i}{{}^{2}}^{1/2}\rangle \;\langle \Delta r_{j}{{}^{2}}^{1/2}\rangle }$$

In the equation, $c_{ij}$ is defined as $c_{ij}=\langle \Delta r_{i}\;\cdot \;\Delta r_{j}\rangle$, where $\Delta r_{i}$ is a displacement vector of atom *i* and $\Delta r_{j}$ of atom *j* and the angle brackets denote an ensemble average. We can obtain the cross-correlation coefficient $C_{ij}$ by normalizing the covariances as shown above. We used PyMOL for additional 3D visualizations of the anticorrelated motions corresponding to the residues in the matrices. 

#### 4.10.4. MM/GBSA Binding Free Energy Calculations

Binding free energy calculations of the protein-ligand complex were performed using the Molecular Mechanics/Generalized Born Surface Area (MM/GBSA) method included in Amber 20 software suit [81,82]. Calculations were performed on 334 snapshots from the last 60 ns of the MD simulation where the simulated system was fully equilibrated. We used the Generalized Born IGB method 5, and a 0.10 M salt concentration. We also performed per-residue decomposition to evaluate the energy contributions of residues to binding.

## Figures and Tables

**Table 1:** Results of the topo IIα HTS relaxation assay of the flavonoid hit compounds **1**–**8**.

Compound	Structure	IC_50_ [µM]
**1**	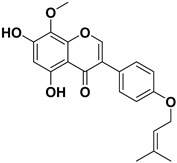	>1000
**2**	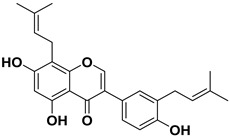	>1000
**3**	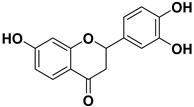	124.7
**4**	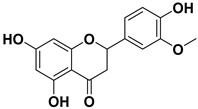	>1000
**5**	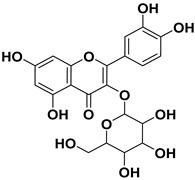	19.4
**6**	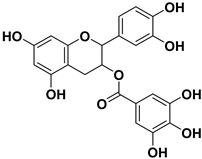	1.7
**7**	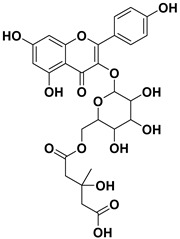	3.9
**8**	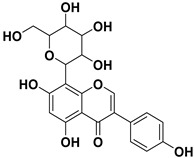	>1000

**Table 2:** The percent of average ATPase activity obtained from two runs for the investigated compounds **6** and **7**, and for etoposide at 4 concentrations.

Average Percentage ATPase Activity				
[etoposide] µM	250	100	50	25
Percentage activity	17.4	52.6	69.7	79.9
[compound **6**] µM	10	5	0.5	0.05
Percentage activity	56.0	60.3	68.2	101.9
[compound **7**] µM	10	5	0.5	0.05
Percentage activity	58.3	69.4	82.5	108.4

## Data Availability

Molecular docking was performed using program Gold (Cambridge Crystallographic Data Centre (CCDC), Cambridge, UK). Results were further visualized in LigandScout, version 4.4.3 (Inte:Ligand, Vienna, Austria). Molecular simulations, analysis and visualization were performed with broadly used programs freely available for academic institutions, Amber, AmberTools 18, VMD 1.9.3 and PyMOL 2.0. Starting structure was obtained from the Protein Data Bank (PDB). All procedures and workflows are described in the Methods Section. Further data of this study are available from the corresponding author upon reasonable request.

## Supplementary Materials

The following are available online at https://www.mdpi.com/article/10.3390/ijms222413474/s1.
