# Supplementary material for: Dynophore-Based Approach in Virtual Screening: A Case of Human DNA Topoisomerase IIα
Source: Int J Mol Sci. 2021 Dec 15;22(24):13474. doi: 10.3390/ijms222413474 (PMC8703789; doi:10.3390/ijms222413474)
Supplement: Supplementary file 1 [file ijms-22-13474-s001.zip › Supplementary Material.pdf]

Supporting information:

Dynophore-based approach in virtual screening: *A case of human DNA topoisomerase IIα*

Matej Janežič[1,2]†, Katja Valjavec[1]†, Kaja Bergant Loboda[1,3], Barbara Herlah[1,3], Iza Ogris[1,4], Mirijam Kozorog[1], Marjetka Podobnik[1], Simona Golič Grdadolnik[1], Gerhard Wolber[5] and Andrej Perdih[1,3]*

[1]National Institute of Chemistry, Hajdrihova 19, SI 1000 Ljubljana, Slovenia

[2]Laboratory for Structural Bioinformatics, RIKEN Center for Biosystems Dynamics Research, 1-7-22 Suehiro-cho, Tsurumi-ku, Yokohama, Kanagawa, 230-0045, Japan

[3]University of Ljubljana, Faculty of Pharmacy, Aškerčeva 7, SI 1000 Ljubljana, Slovenia

[4]University of Ljubljana, Faculty of Medicine, Vrazov trg 2, 1000 Ljubljana, Slovenia

[5]Institute of Pharmacy, Freie Universität Berlin, Königin Luise-Strasse 2+4, 14195 Berlin, Germany

[†] *These two authors contributed equally to this work.*

Corresponding author:

National Institute of Chemistry

Hajdrihova 19, SI-1001 Ljubljana, Slovenia

E-mail : andrej.perdih@ki.si

Tel.: +386-1-4760-376

1. Dynophores models of the initial AMP-PNP ligand and triazinone catalytic inhibitor

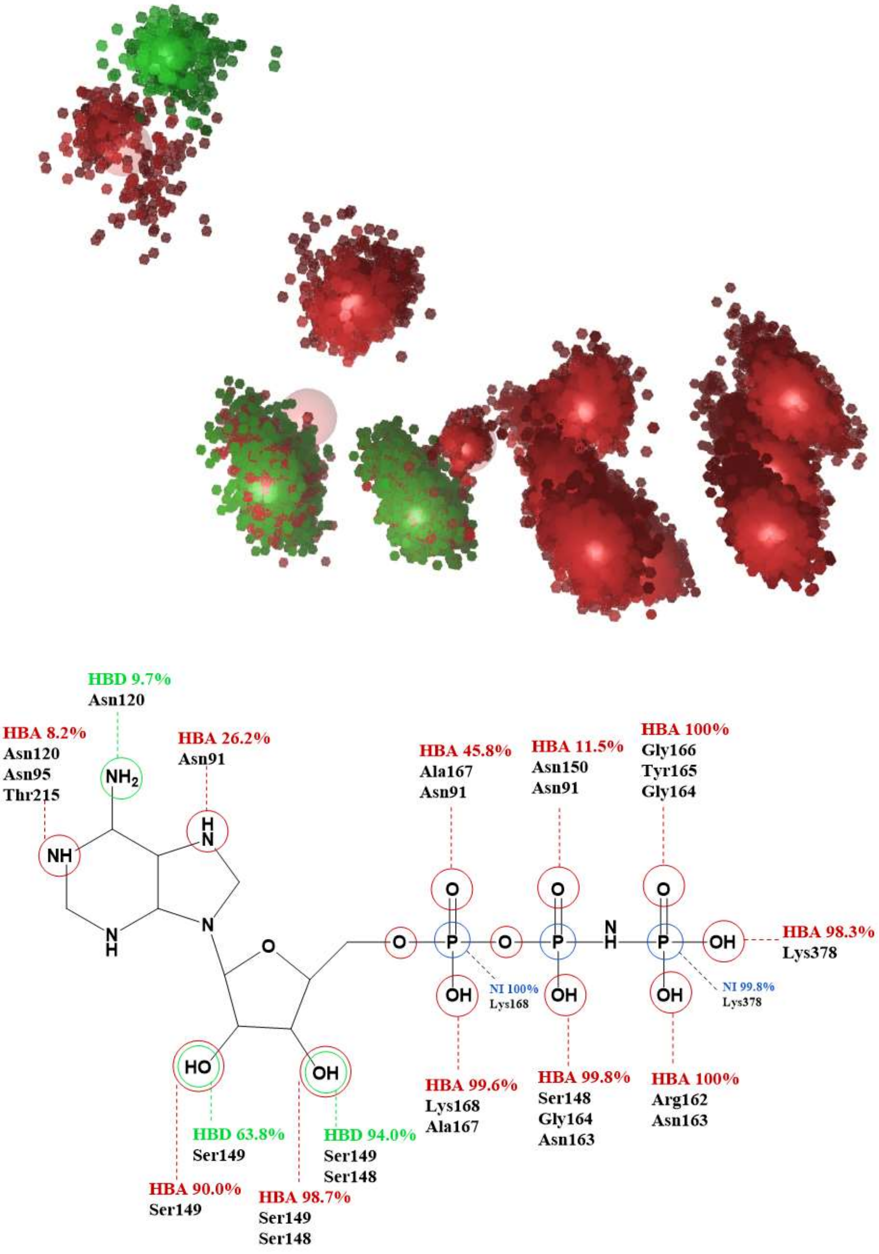

Figure S1. (Top) Dynophore model of the AMP-PNP ligand. (Bellow) Detailed interaction pattern of the obtained dynophore model.

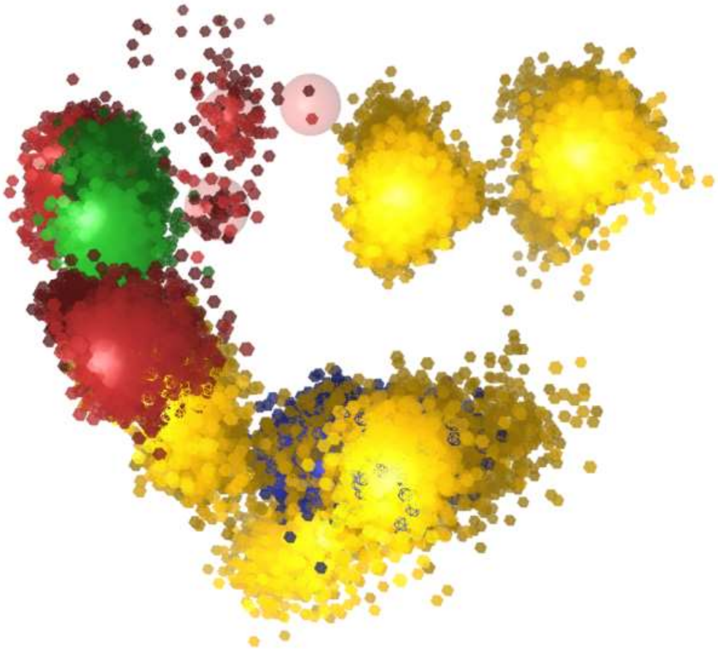

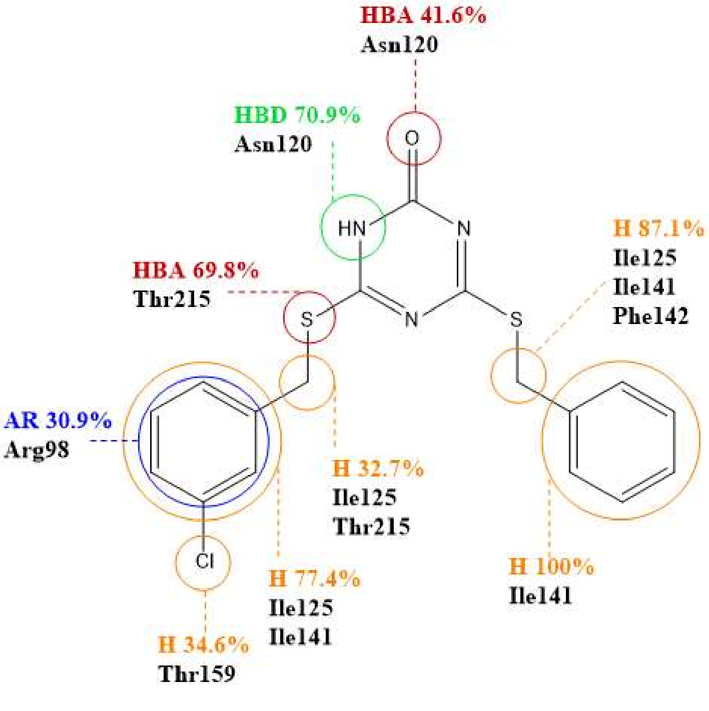

Figure S2. (Top) Dynophore model of the triazinone catalytic inhibitor. (Bellow) Detailed interaction pattern of the derived dynophore model.

2. Pharmacophore-based virtual screening

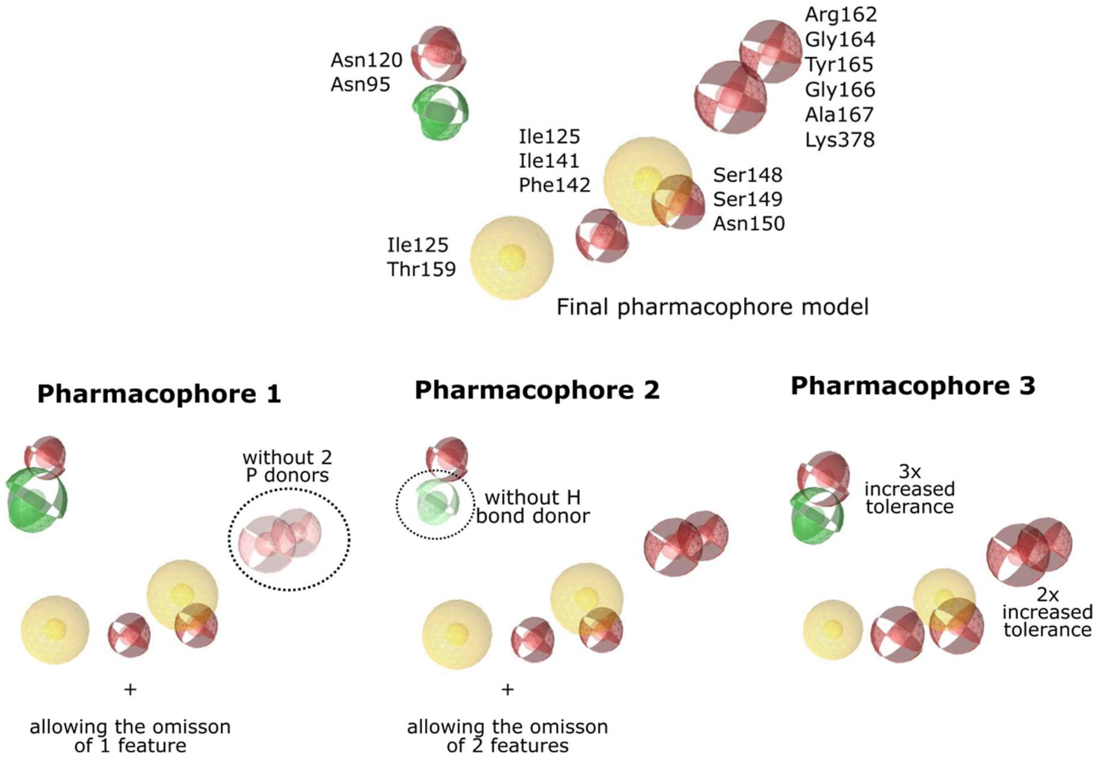

Figure S3. Master pharmacophore model derived from the joint dynophore model and the three submodels (pharmacophore models 1-3) that were used in the virtual screening campaign.

3. List of assayed compounds and their vendor IDs

Table S1. 2D chemical structures and vendor IDs of the assayed hit compounds identified in the pharmacophore-based virtual screening campaign. All compounds were supplied by AnalytiCon Discovery.

Number	Vendor id	Chemical structure
**1**	NP-020233	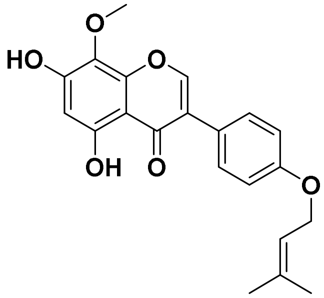
**2**	NP-012878	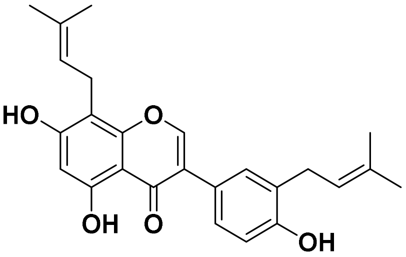
**3**	NP-000008	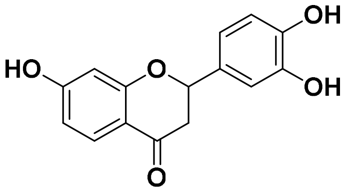
**4**	NP-001581	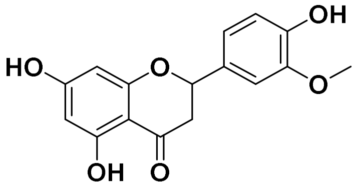
**5**	NP-001009	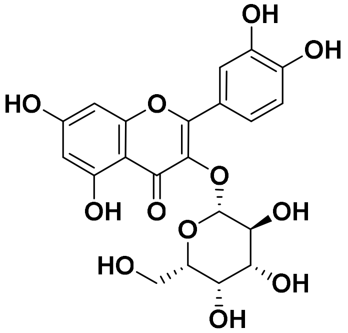

**6**	NP-002326	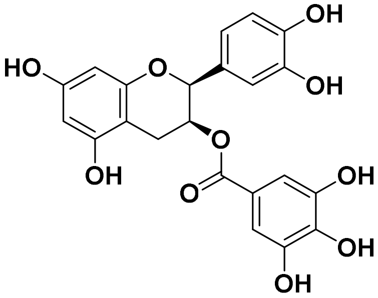
**7**	NP-018721	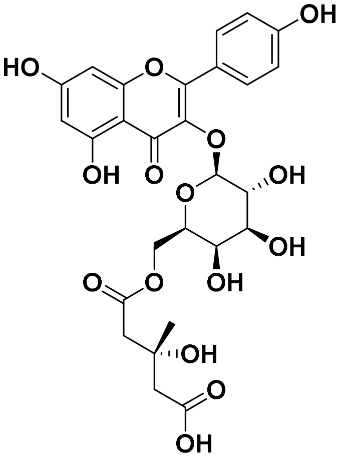
**8**	NP-002406	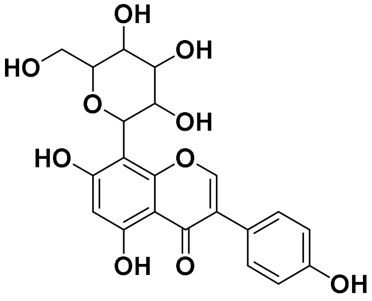
**9**	NP-021038	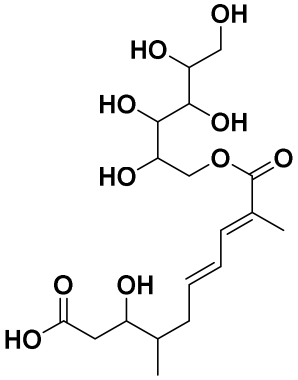
**10**	NP-003468	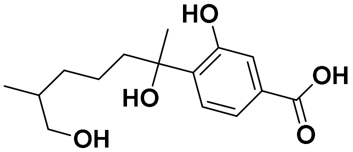

**11**	NP-015334	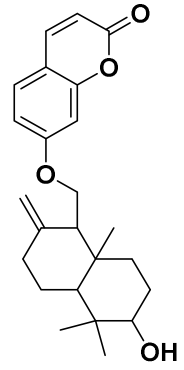
**12**	NP-021204	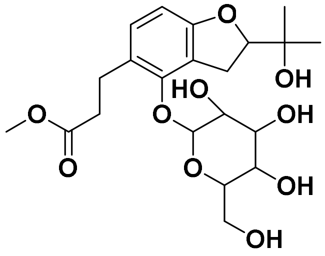
**13**	NP-015244	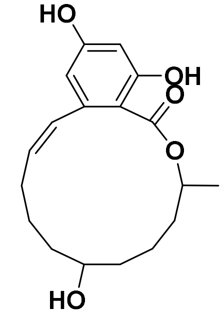
**14**	NP-009245	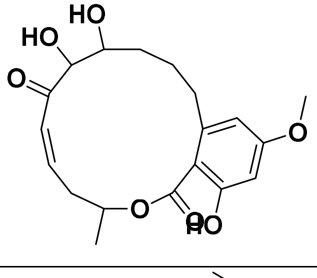
**15**	NP-008285	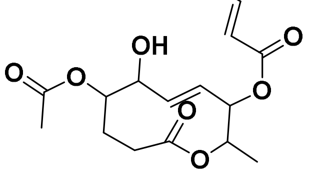
**16**	NP-004128	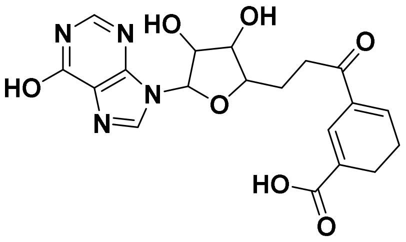

**16**	NAT12-540804	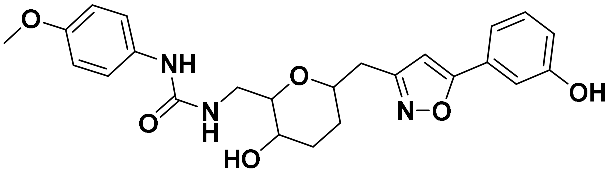
**18**	NAT12-540805	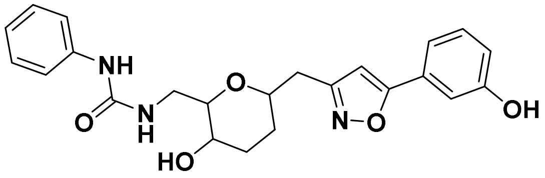
**19**	NAT13-337482	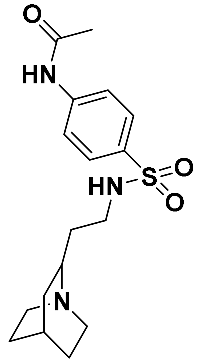
**20**	NAT13-332258	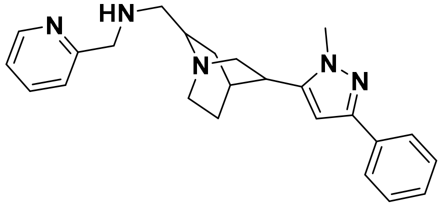
**21**	NAT14-324144	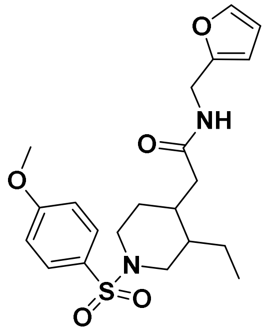
**22**	NAT14-324143	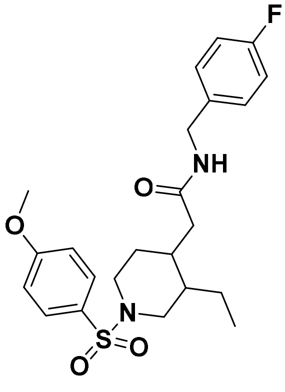

**23**	NAT16-353105	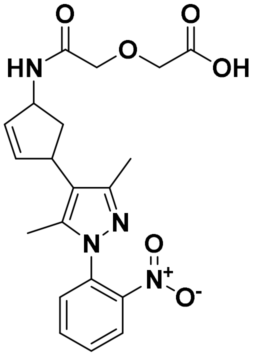
**24**	NAT19-353700	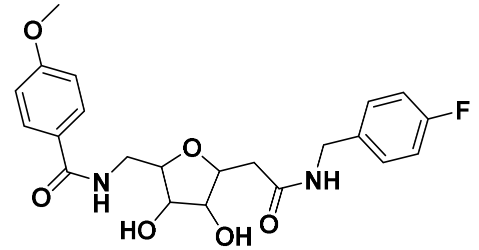
**25**	NAT19-353596	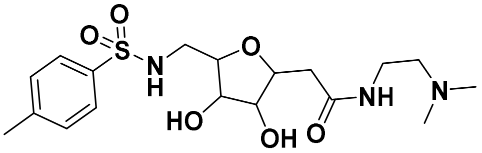
**26**	NAT217-567834	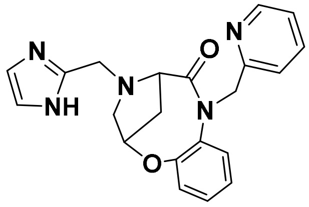
**27**	NAT225-569709	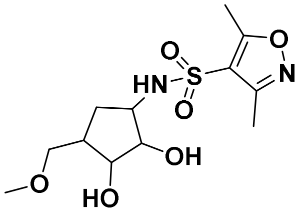
**28**	NAT231-583844	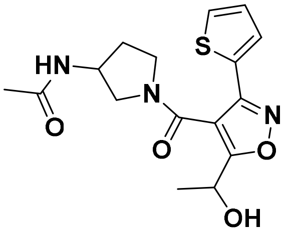

**29**	NAT231-583923	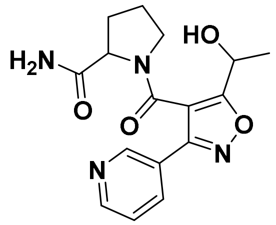
**30**	NAT231-584163	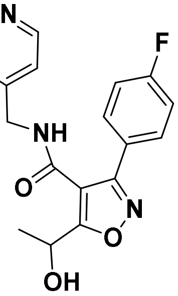
**31**	NAT231-584167	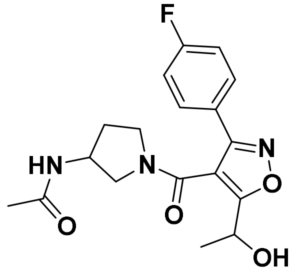
**32**	NAT231-584289	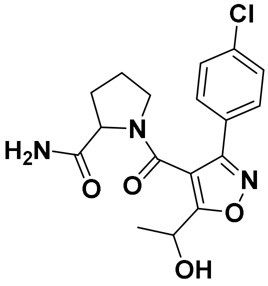
**33**	NAT231-584332	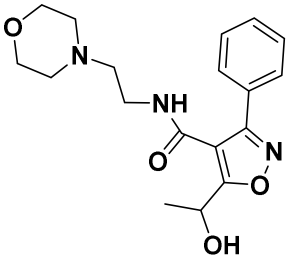
**34**	NAT231-584350	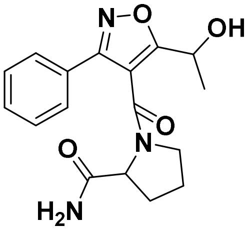

**35**	NAT28-409157	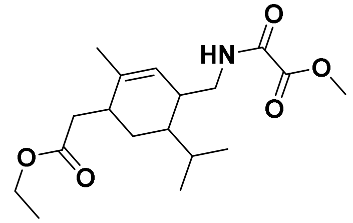
**36**	NAT31-456614	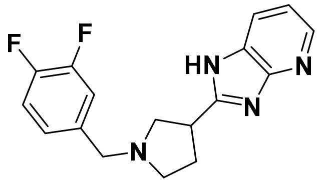
**37**	NAT31-460146	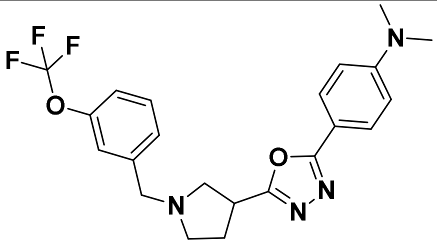
**38**	NAT33-501383	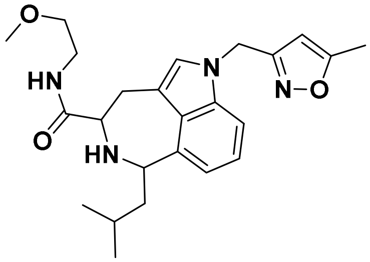
**39**	NAT33-501388	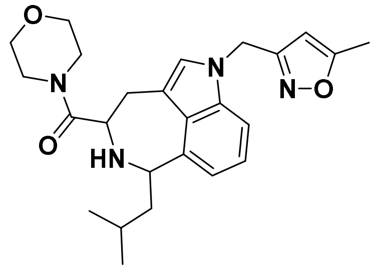
**40**	NAT36-504794	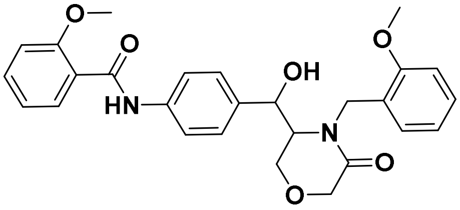
**41**	NAT36-530837	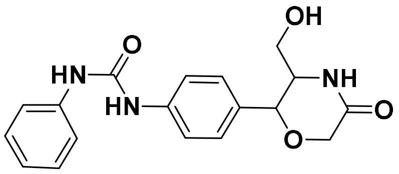

**42**	NAT36-530845	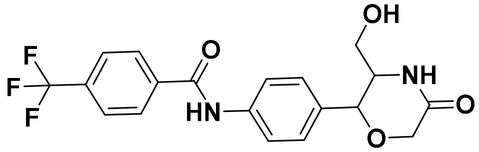
**43**	NAT36-530847	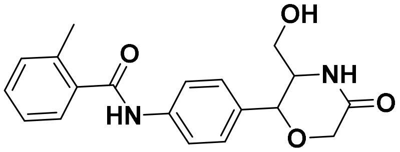
**44**	NAT39-500350	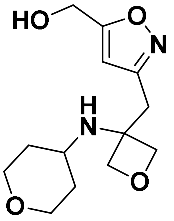
**45**	NAT39-500427	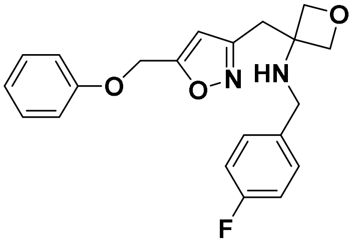
**46**	NAT49-563038	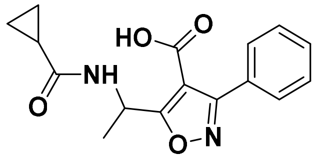
**47**	NAT5-250415	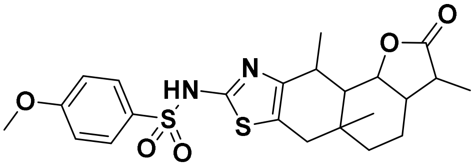
**48**	NAT50-555957	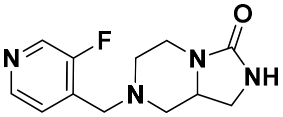
**49**	NAT9-312632	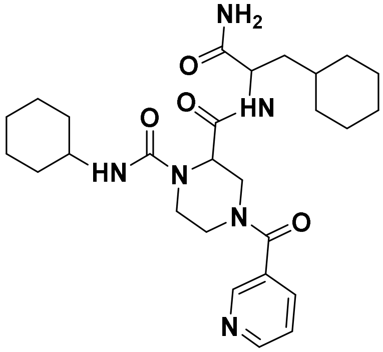

4. Results of the human topo IIα decatenation assay

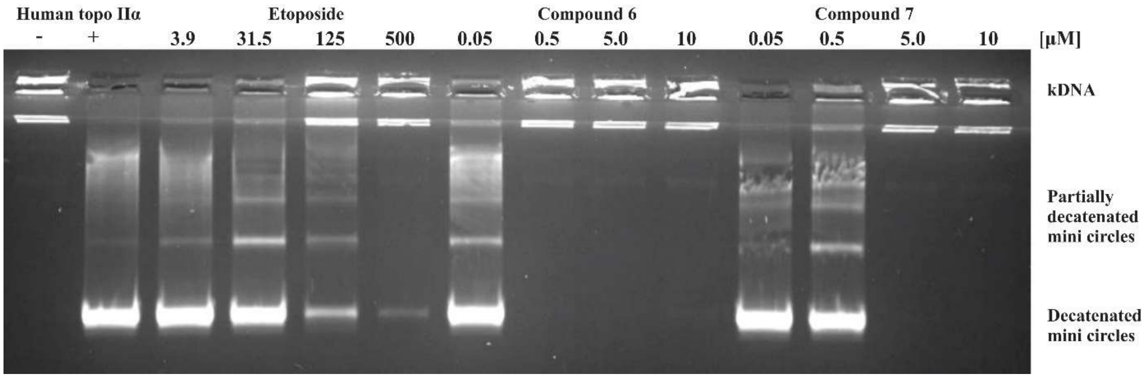

Figure S4. Results of the second run of the of the human topo IIα decatenation assay for compounds **6**, **7**, and etoposide. The assay was performed at 4 different concentrations (0.05, 0.5, 5.0 and 10 μM) of compound **6**, and **7**. For the reference compound etoposide, concentrations 3.9, 31.5, 125, 500 μM were used.

Table S2. Detailed data of the decatenation assay catalysed by the human topo IIα for compounds **6** and **7**, and positive control etoposide. Results are represented as % of the decatenated kDNA.

**Compound**	**% Decatenated Assay 1**				**% Decatenated Assay 2**				**% Decatenated Average**			
**Concentration (μM)**	**3.9**	**31.5**	**125**	**500**	**3.9**	**31.5**	**125**	**500**	**3.9**	**31.5**	**125**	**500**
**Etoposide**	100.0	92.4	28.9	4.9	99.9	87.4	25.9	5.2	100	89.9	27.4	5.0
**Concentration (μM)**	**0.05**	**0.5**	**5.0**	**10**	**0.05**	**0.5**	**5.0**	**10**	**0.05**	**0.5**	**5.0**	**10**
**Compound 6**	41.1	0.15	0	0	88.3	0	0	0	64.7	0.08	0	0
**Concentration (μM)**	**0.05**	**0.5**	**5.0**	**10**	**0.05**	**0.5**	**5.0**	**10**	**0.05**	**0.5**	**5.0**	**10**
**Compound 7**	98.0	98.6	0	0	99.2	89.1	0	0	98.6	93.9	0	0

5. Results of the human topo IIα cleavage assay

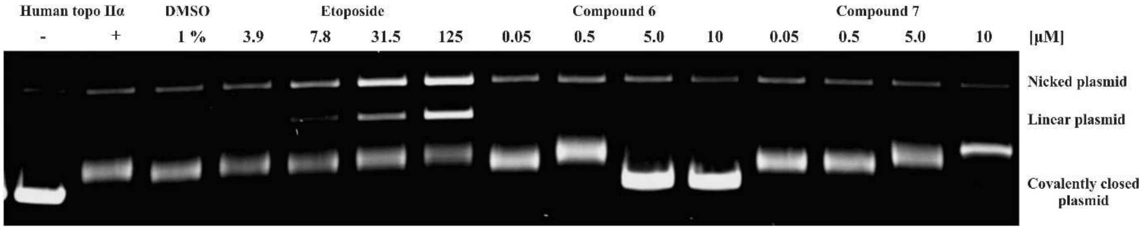

Figure S5. Results of the second run of the topoisomerase IIα cleavage assay. The assay was performed at 4 different concentrations 0.05, 0.5, 5.0 and 10 μM of compound **6** and **7** and at 3.9, 7.8, 31.5, 125 μM concentrations of the reference compound etoposide. The linear band was measured as a % of the total DNA, with the 100% representing the amount of DNA in track 1 (DNA alone).

Table S3. Percent of the linear DNA determined in the topoisomerase IIα cleavage assay for compounds **6** and **7** and etoposide as a reference molecule (all measured at four concentrations).

**Compound**	**% Linear Assay 1**	**% Linear Assay 2**	**% Linear Average**
DNA alone	0	0	**0**
DNA + topo II	0	0	**0**
DNA + topo II + DMSO	0	1.80	**0.90**
Etoposide 3.9 μM	10.31	5.52	**7.92**
Etoposide 7.8 μM	13.40	10.07	**11.73**
Etoposide 31.5 μM	21.43	16.90	**19.16**
Etoposide 125 μM	35.87	31.47	**33.67**
Compound **6** 0.05 μM	0	0	**0**
Compound **6** 0.5 μM	0	0	**0**
Compound **6** 5.0 μM	0	0	**0**
Compound **6** 10 μM	0	0	**0**
Compound **7** 0.05 μM	0	0	**0**
Compound **7** 0.5 μM	0	0	**0**
Compound **7** 5.0 μM	0	0	**0**
Compound **7** 10 μM	0	0	**0**

6. Results of the unwinding assay

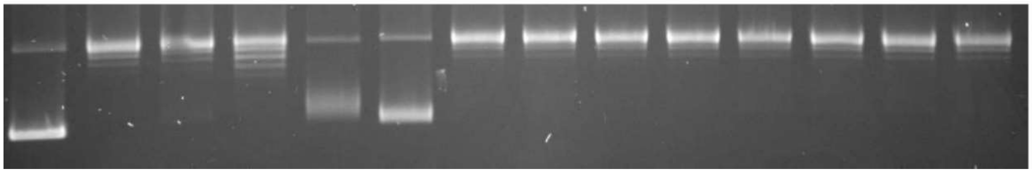

Figure S6. Results of the second run of the unwinding assay. The assay was performed at four different concentrations of compounds **6** and **7** (0.05, 0.5, 5.0 and 10 μM) and of the positive control intercalator mAMSA (5.0, 10, 50, 100 μM).

7. Results of the human topo IIα ATPase activity

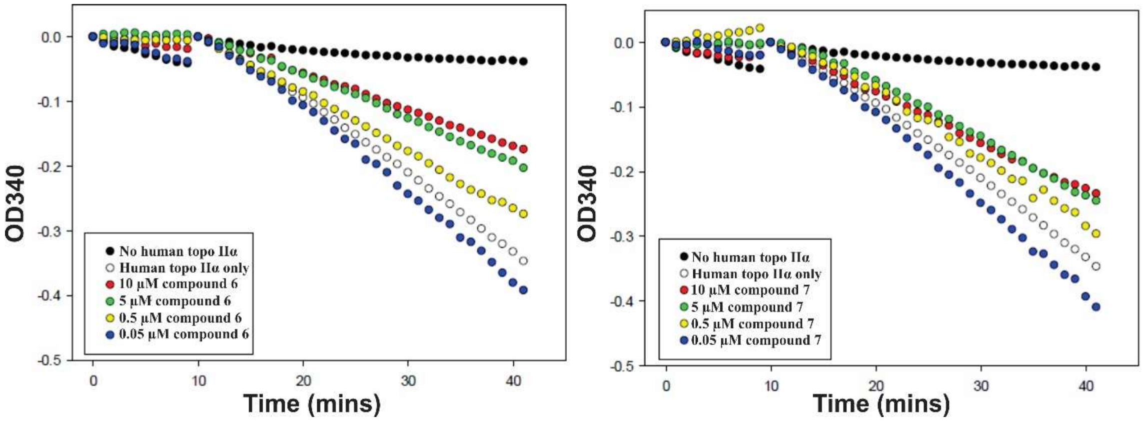

Figure S7. Representative graphs of the human topo IIα ATPase assay for compounds **6** (left) and **7** (right). The reaction was performed at four concentrations (0.05, 0.5, 5.0 and 10 µM) for each investigated compound.

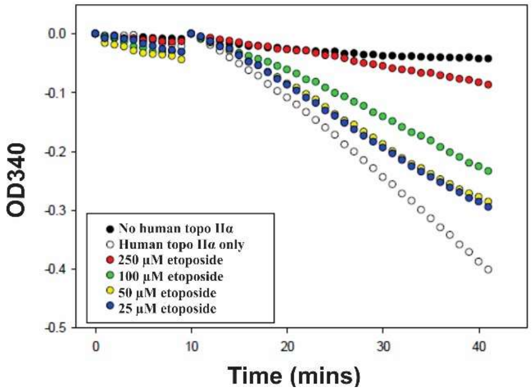

Figure S8. Representative graph of the performed ATPase assay for the etoposide reference molecule. The reaction was performed at four concentrations (25, 50, 100, 250 µM).

Table S4. Results of the human topo IIα ATPase assay for compounds **6** and **7,** and etoposide given as the % activity which were calculated as follows: The spectrophotometer produces an output as change in OD340 with time. After 40 or 60 minutes the blank reading (no enzyme) was subtracted from the readings for each compound and divided by the positive reading (no inhibitor, 100% activity) minus the blank to give the percentage ATPase activity for each compound.

	**Rate ATPase activity (μmol/min)**							
	**Run 1**				**Run 2**			
**No human topo IIα**	0.342		0.218		0.342		0.612	
	0.769				0.28		0.363	
**Human topo IIα only**	4.08		4.38		3.63		4.03	
	4.12		3.94		3.86		2.87	
	**Average no human topo IIα**		0.443		**Average no human topo IIα**		0.399	
	**Average human topo IIα only**		4.13		**Average human topo IIα only**		3.60	
	**Percentage ATPase activity of compounds**				**Percentage ATPase activity of compounds**			
	**Concentration (μM) Run 1**				**Concentration (μM) Run 2**			
**[Etoposide]**	**250**	**100**	**50**	**25**	**250**	**100**	**50**	**25**
**Percentage**	14.9	55.1	69.3	71.8	19.9	50.1	70.0	87.9
**[cpd 6]**	**10**	**5**	**0.5**	**0.05**	**10**	**5**	**0.5**	**0.05**
**Percentage**	58.7	48.8	60.0	89.2	43.9	53.3	76.3	114.6
**[cpd 7]**	**10**	**5**	**0.5**	**0.05**	**10**	**5**	**0.5**	**0.05**
**Percentage**	53.2	71.8	81.4	96.4	63.4	66.9	83.6	120.4

**Average Percentage Activity**				
[Etoposide] **μM**	**250**	**100**	**50**	**25**
Percentage activity	17.4	52.6	69.7	79.9
**[cpd 6] μM**	**10**	**5**	**0.5**	**0.05**
Percentage activity	56.0	60.3	68.2	101.9
**[cpd 7] μM**	**10**	**5**	**0.5**	**0.05**
Percentage activity	58.3	69.4	82.5	108.4

8. Results of the competitive cleavage assay for compound 6

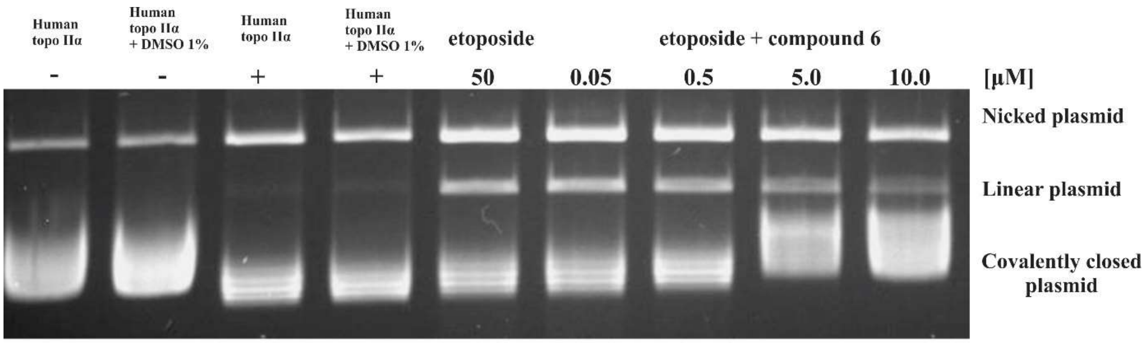

Figure S9. Results of the second run of the topoisomerase IIα competitive cleavage assay. The assay was performed at 4 different concentrations of compound **6** (0.05, 0.5, 5.0 and 10 μM) in the presence of etoposide at the 50 μM concentration.

Table S5. Results of the competitive cleavage assay for compound **6**, tested at four concentrations: 0.05, 0.5, 5.0, 10 μM in the presence of 50 μM etoposide.

**Compound**	**% Linear Assay 1**	**% Linear Assay 2**	**% Linear Average**
DNA alone	0	0	**0**
DNA + topo IIα	1.58	2.08	**1.83**
DNA + topo IIα + DMSO	1.25	2.37	**1.81**
+ Etoposide 50 μM	22.25	17.33	**19.79**
+ Compound **6** 0.05 μM	19.03	17.70	**18.37**
+ Compound **6** 0.5 μM	16.41	16.50	**16.45**
+ Compound **6** 5.0 μM	19.61	8.86	**14.24**
+ Compound **6** 10 μM	6.51	4.74	**5.63**

9. Results of the competitive ATPase assay for compound 6

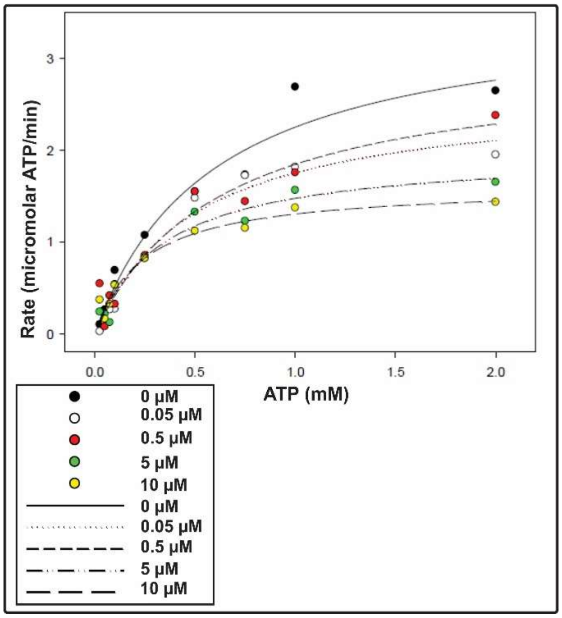

Figure S10. Representative graph of the competitive ATPase assay for compound **6**, performed at 4 different concentrations of compound **6** (0.05, 0.5, 5.0 and 10 μM), and 9 different ATP concentrations (0.025, 0.05, 0.075, 0.1, 0.25, 0.5, 0.75, 1, 2 mM).

10.HR-MS analysis of active compounds

The HR-MS analysis were performed at the Centre for Mass spectroscopy, Josef Stefan Institute, Ljubljana, Slovenia.

Elemental Composition Report — Page 1

Single Mass Analysis
Tolerance = 10.0 PPM / DBE: min = -1.5, max = 50.0
Element prediction: Off
Number of isotope peaks used for i-FIT = 3

Compound 3

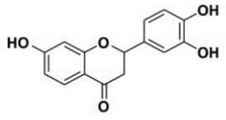

Monoisotopic Mass, Even Electron Ions
471 formula(e) evaluated with 5 results within limits (all results (up to 1000) for each mass)
Elements Used:
C: 0-100 H: 0-100 N: 0-20 O: 0-20
Kaja Bergant
NP-000008 neg 30 (1.219) Cm (29:31-3:10)

1: TOF MS ES-
1.97e+005

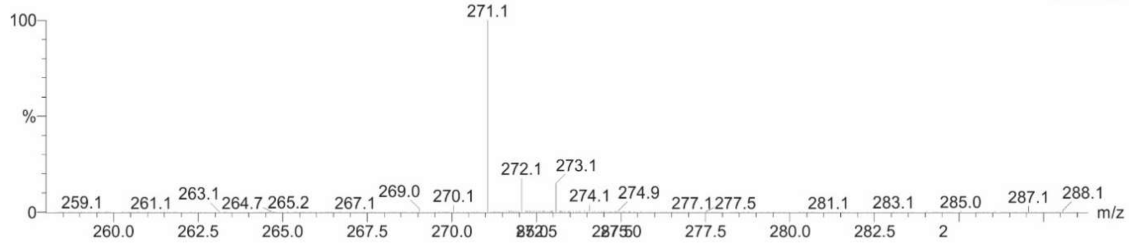

Minimum: -1.5
Maximum: 5.0 10.0 50.0

Mass	Calc. Mass	mDa	PPM	DBE	i-FIT	i-FIT (Norm)	Formula
271.0613	271.0612	0.1	0.4	3.5	617.8	2.8	H7 N12 O6
	271.0606	0.7	2.6	10.5	615.9	0.9	C15 H11 O5
	271.0620	-0.7	-2.6	15.5	616.0	1.0	C16 H7 N4 O
	271.0625	-1.2	-4.4	8.5	618.4	3.3	C H3 N16 O2
	271.0638	-2.5	-9.2	2.5	617.3	2.3	C4 H11 N6 O8

Elemental Composition Report

Single Mass Analysis

Tolerance = 10.0 PPM / DBE: min = -1.5, max = 50.0
Element prediction: Off
Number of isotope peaks used for i-FIT = 3

Compound 5

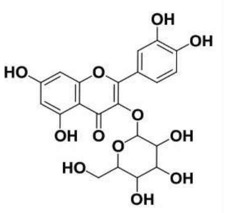

Monoisotopic Mass, Even Electron Ions
1539 formula(e) evaluated with 20 results within limits (all results (up to 1000) for each mass)
Elements Used:
C: 0-100 H: 0-100 N: 0-20 O: 0-20
Kaja Bergant
NP-001009 neg 41 (1.680) Cm (41:42)

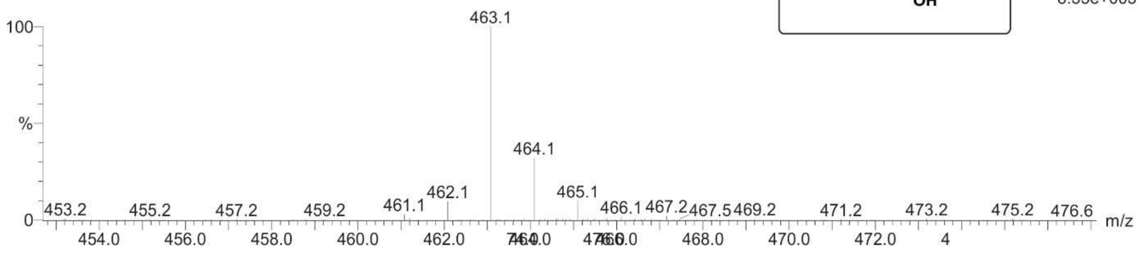

Minimum: -1.5
Maximum: 5.0 10.0 50.0

Mass	Calc. Mass	mDa	PPM	DBE	i-FIT	i-FIT (Norm)	Formula
463.0873	463.0871	0.2	0.4	30.5	176.1	1.2	C34 H11 N2 O
	463.0876	-0.3	-0.6	23.5	178.8	3.9	C19 H7 N14 O2
	463.0877	-0.4	-0.9	12.5	177.7	2.8	C21 H19 O12
	463.0868	0.5	1.1	0.5	182.0	7.0	C5 H19 N8 O17
	463.0882	-0.9	-1.9	5.5	181.9	6.9	C6 H15 N12 O13
	463.0863	1.0	2.2	18.5	178.9	3.9	C18 H11 N10 O6
	463.0890	-1.7	-3.7	17.5	177.6	2.6	C22 H15 N4 O8
	463.0855	1.8	3.9	6.5	183.0	8.0	C2 H11 N18 O11
	463.0895	-2.2	-4.8	-0.5	181.0	6.1	C9 H23 N2 O19
	463.0895	-2.2	-4.8	10.5	181.8	6.8	C7 H11 N16 O9
	463.0850	2.3	5.0	13.5	179.0	4.1	C17 H15 N6 O10
	463.0850	2.3	5.0	24.5	180.1	5.1	C15 H3 N20
	463.0903	-3.0	-6.5	22.5	177.6	2.6	C23 H11 N8 O4
	463.0841	3.2	6.9	1.5	183.3	8.3	C H15 N14 O15
	463.0908	-3.5	-7.6	4.5	180.9	6.0	C10 H19 N6 O15
	463.0908	-3.5	-7.6	15.5	181.7	6.7	C8 H7 N20 O5
	463.0836	3.7	8.0	19.5	180.2	5.2	C14 H7 N16 O4
	463.0836	3.7	8.0	8.5	179.3	4.3	C16 H19 N2 O14
	463.0831	4.2	9.1	26.5	176.1	1.1	C29 H11 N4 O3
	463.0917	-4.4	-9.5	27.5	177.8	2.8	C24 H7 N12

Elemental Composition Report

Compound 6

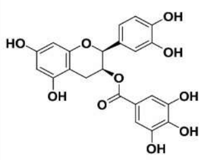

Single Mass Analysis

Tolerance = 10.0 PPM / DBE: min = -1.5, max = 50.0
Element prediction: Off
Number of isotope peaks used for i-FIT = 3

Monoisotopic Mass, Even Electron Ions
1419 formula(e) evaluated with 15 results within limits (all results (up to 1000) for each mass)
Elements Used:
C: 0-100 H: 0-100 N: 0-20 O: 0-20
Kaja Bergant
NP-002326 15 (0.609) Cm (13:16)

1: TOF MS ES+
7.97e+003

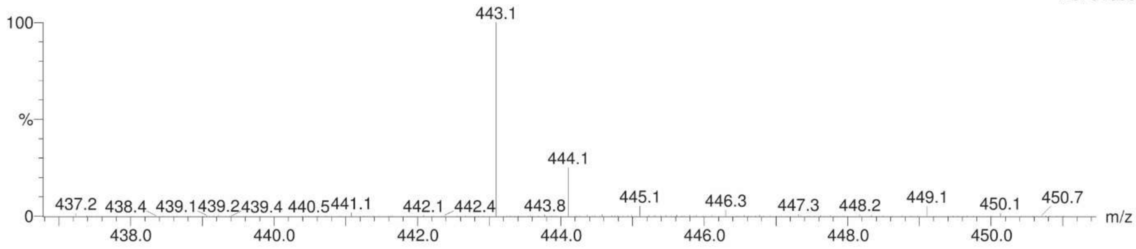

Mass	Calc. Mass	mDa	PPM	DBE	i-FIT	i-FIT (Norm)	Formula
Minimum:				-1.5			
Maximum:		5.0	10.0	50.0			
443.0984	443.0983	0.1	0.2	6.5	218.4	13.2	C7 H15 N12 O11
	443.0978	0.6	1.4	13.5	205.2	0.0	C22 H19 O10
	443.0978	0.6	1.4	24.5	212.6	7.5	C20 H7 N14
	443.0992	-0.8	-1.8	18.5	209.1	3.9	C23 H15 N4 O6
	443.0997	-1.3	-2.9	0.5	215.4	10.3	C10 H23 N2 O17
	443.0997	-1.3	-2.9	11.5	218.1	12.9	C8 H11 N16 O7
	443.0970	1.4	3.2	1.5	218.7	13.6	C6 H19 N8 O15
	443.0965	1.9	4.3	19.5	211.3	6.2	C19 H11 N10 O4
	443.1005	-2.1	-4.7	23.5	212.4	7.2	C24 H11 N8 O2
	443.1010	-2.6	-5.9	16.5	217.9	12.8	C9 H7 N20 O3
	443.1010	-2.6	-5.9	5.5	216.0	10.8	C11 H19 N6 O13
	443.0956	2.8	6.3	7.5	221.3	16.1	C3 H11 N18 O9
	443.0951	3.3	7.4	14.5	211.9	6.8	C18 H15 N6 O8
	443.1023	-3.9	-8.8	10.5	216.2	11.1	C12 H15 N10 O9
	443.0943	4.1	9.3	2.5	222.0	16.8	C2 H15 N14 O13

Elemental Composition Report

Page 1

Compound 7

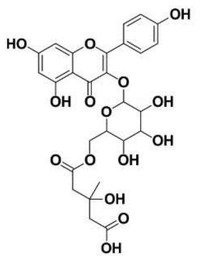

Single Mass Analysis

Tolerance = 10.0 PPM / DBE: min = -1.5, max = 50.0
Element prediction: Off
Number of isotope peaks used for i-FIT = 3

Monoisotopic Mass, Even Electron Ions
2314 formula(e) evaluated with 29 results within limits (all results (up to 1000) for each mass)
Elements Used:
C: 0-100 H: 0-100 N: 0-20 O: 0-20
Kaja Bergant
NP-018721 21 (0.868) Cm (18:21)

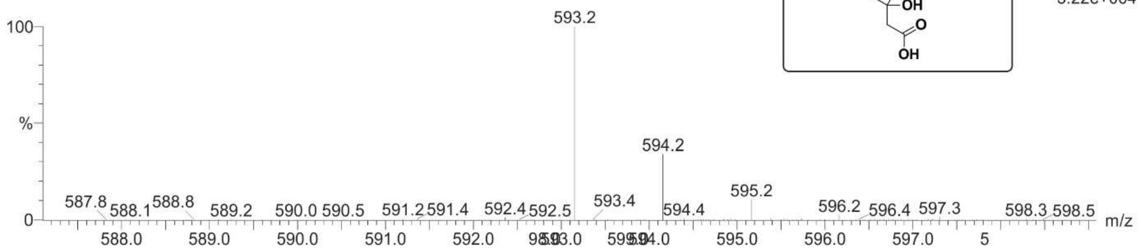

Minimum: -1.5
Maximum: 5.0 10.0 50.0

Mass	Calc. Mass	mDa	PPM	DBE	i-FIT	i-FIT (Norm)	Formula
593.1505	593.1506	-0.1	-0.2	13.5	227.0	1.7	C27 H29 O15
	593.1506	-0.1	-0.2	24.5	229.2	4.0	C25 H17 N14 O5
	593.1501	0.4	0.7	31.5	229.0	3.8	C40 H21 N2 O4
	593.1511	-0.6	-1.0	6.5	233.1	7.9	C12 H25 N12 O16
	593.1498	0.7	1.2	1.5	233.4	8.2	C11 H29 N8 O20
	593.1515	-1.0	-1.7	36.5	229.8	4.6	C41 H17 N6
	593.1493	1.2	2.0	19.5	229.2	3.9	C24 H21 N10 O9
	593.1520	-1.5	-2.5	29.5	229.6	4.4	C26 H13 N18 O
	593.1520	-1.5	-2.5	18.5	226.9	1.7	C28 H25 N4 O11
	593.1525	-2.0	-3.4	11.5	233.0	7.8	C13 H21 N16 O12
	593.1485	2.0	3.4	7.5	234.6	9.3	C8 H21 N18 O14
	593.1480	2.5	4.2	14.5	229.5	4.3	C23 H25 N6 O13
	593.1479	2.6	4.4	25.5	231.1	5.9	C21 H13 N20 O3
	593.1533	-2.8	-4.7	23.5	227.5	2.3	C29 H21 N8 O7
	593.1474	3.1	5.2	32.5	229.3	4.1	C36 H17 N8 O2
	593.1538	-3.3	-5.6	5.5	231.8	6.5	C16 H29 N6 O18
	593.1538	-3.3	-5.6	16.5	232.9	7.6	C14 H17 N20 O8
	593.1471	3.4	5.7	2.5	234.9	9.6	C7 H25 N14 O18
	593.1542	-3.7	-6.2	35.5	230.0	4.7	C45 H21 O2
	593.1543	-3.8	-6.4	-1.5	236.4	11.1	C H25 N18 O19
	593.1466	3.9	6.6	20.5	231.3	6.1	C20 H17 N16 O7
	593.1466	3.9	6.6	9.5	230.1	4.8	C22 H29 N2 O17
	593.1547	-4.2	-7.1	28.5	228.5	3.3	C30 H17 N12 O3
	593.1461	4.4	7.4	27.5	228.5	3.2	C35 H21 N4 O6
	593.1552	-4.7	-7.9	10.5	231.6	6.4	C17 H25 N10 O14
	593.1453	5.2	8.8	15.5	231.6	6.4	C19 H21 N12 O11
	593.1560	-5.5	-9.3	22.5	226.9	1.6	C33 H25 N2 O9
	593.1448	5.7	9.6	33.5	230.0	4.7	C32 H13 N14
	593.1448	5.7	9.6	22.5	227.4	2.2	C34 H25 O10

11. Molecular docking calculations

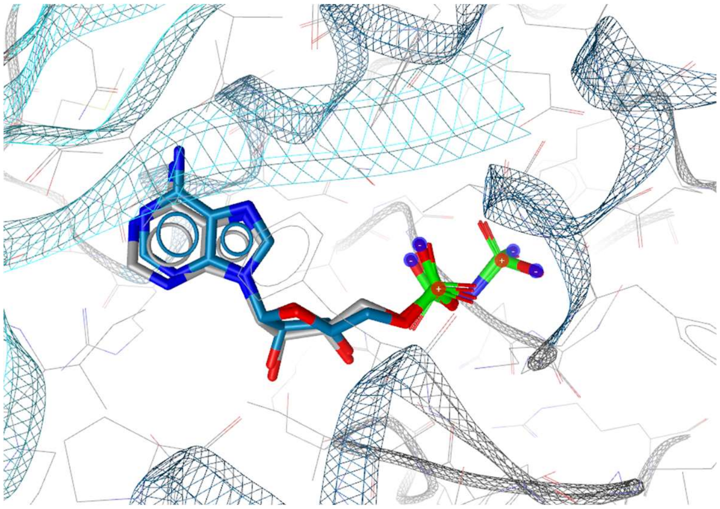

Figure S11. Comparison of the docked AMP-PNP ligand (gray) and its x-ray conformation (blue) in the ATP binding site of the human topo IIα (PDB: 1ZXM, chain A). A RMSD of 0.9 Å between heavy atoms of both poses was determined.

12. Partial charges and Atoms Types of the simulated Compound 6

Table S6. Atom types and partial atomic charges for compound **6** generated using General Amber Force Field (gaff).

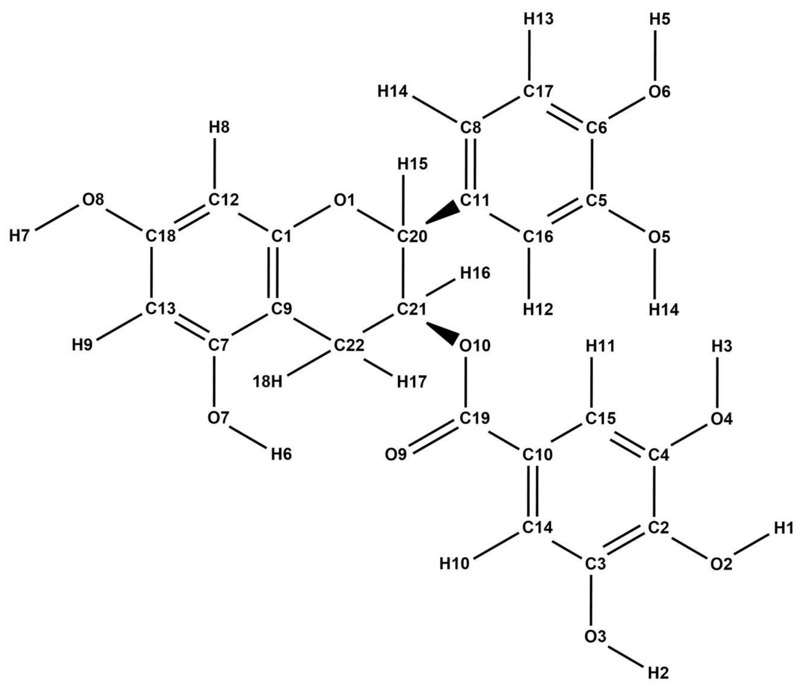

Name	Type	q	Name	Type	q
**O5**	oh	-0.661984	**H9**	ha	0.275341
**H4**	ho	0.470859	**H8**	ha	0.23262
**C5**	ca	0.234378	**C9**	ca	-0.50207
**C16**	ca	-0.315206	**C22**	c3	0.019471
**H12**	ha	0.176945	**H17**	hc	0.051225
**C6**	ca	0.37889	**H18**	hc	0.051225
**O6**	oh	-0.641321	**C21**	c3	0.270078
**H5**	ho	0.471277	**H16**	h1	0.067092
**C17**	ca	-0.309891	**O10**	os	-0.593503

**H13**	ha	0.218938	**C19**	c	1.045.982
**C18**	ca	-0.242514	**O9**	o	-0.662182
**H14**	ha	0.171712	**C10**	ca	-0.254459
**C11**	ca	0.031387	**C15**	ca	-0.322011
**C20**	c3	0.236837	**H11**	ha	0.232831
**H15**	h1	0.072468	**C14**	ca	-0.322011
**O1**	os	-0.431101	**H10**	ha	0.232831
**C1**	ca	0.510958	**C3**	ca	0.374428
**C12**	ca	-0.670155	**O3**	oh	-0.61413
**C8**	ca	0.603985	**H2**	ho	0.446264
**O8**	oh	-0.640153	**C2**	ca	0.062867
**H7**	ho	0.449332	**O2**	oh	-0.590765
**C13**	ca	-0.618825	**H1**	ho	0.415796
**C7**	ca	0.570294	**C4**	ca	0.374428
**O7**	oh	-0.634612	**O4**	oh	-0.61413
**H6**	ho	0.44402	**H3**	ho	0.446264

13. Analysis of the MD trajectory of the topo IIα - compound 6 complex

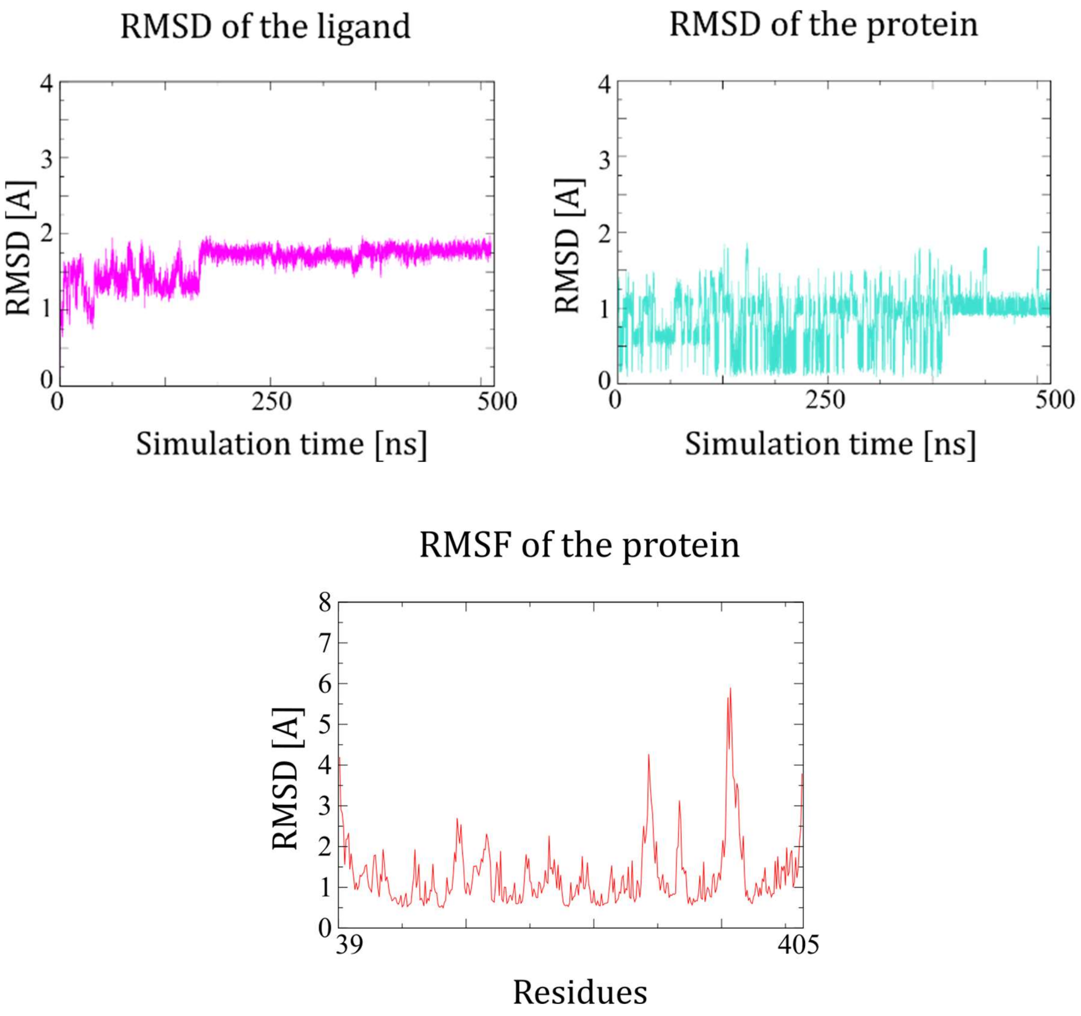

Figure S12. (left) Time-dependant RMSD of the ligand **6** (right), Time-dependant RMSD of the protein (Cα atoms) and (bellow) RMSF graph of the protein during the MD simulation.

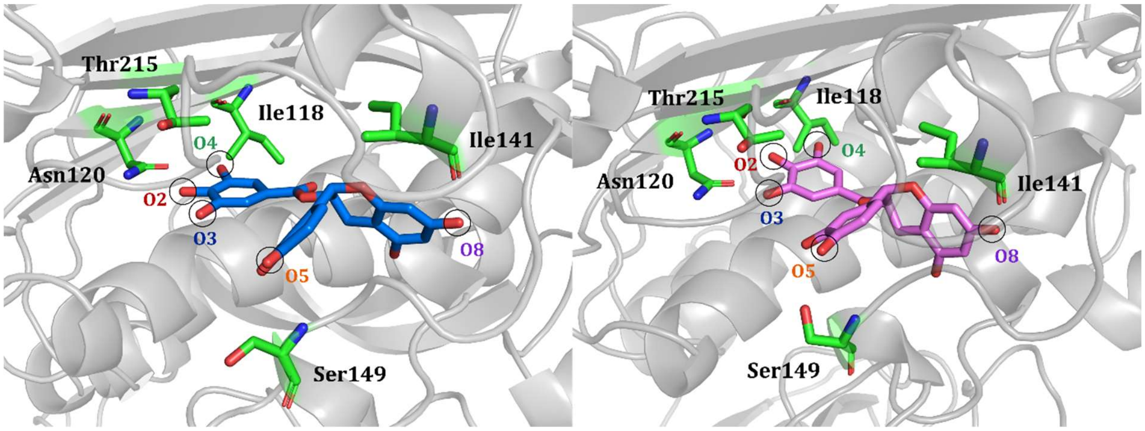

Figure S13. Positional shift of ligand **6** in the ATP binding site during the MD simulation. (Left) Position during the first 150 ns. (Right) Position from 150 ns onward.
